# A "White" Anthocyanin-less Pomegranate (*Punica granatum* L.) Caused by an Insertion in the Coding Region of the Leucoanthocyanidin Dioxygenase (*LDOX; ANS*) Gene

**DOI:** 10.1371/journal.pone.0142777

**Published:** 2015-11-18

**Authors:** Zohar Ben-Simhon, Sylvie Judeinstein, Taly Trainin, Rotem Harel-Beja, Irit Bar-Ya'akov, Hamutal Borochov-Neori, Doron Holland

**Affiliations:** 1 Unit of Deciduous Fruit Tree Sciences, Newe Ya’ar Research Center, Agricultural Research Organization, P.O. Box 1021, Ramat Yishay, 30095, Israel; 2 Faculty of Biology, Technion- Israel Institute of Technology, Haifa, Israel; 3 Southern Arava Research and Development, Hevel Eilot, 88820, Israel; Zhejiang University, CHINA

## Abstract

Color is an important determinant of pomegranate fruit quality and commercial value. To understand the genetic factors controlling color in pomegranate, chemical, molecular and genetic characterization of a "white" pomegranate was performed. This unique accession is lacking the typical pomegranate color rendered by anthocyanins in all tissues of the plant, including flowers, fruit (skin and arils) and leaves. Steady-state gene-expression analysis indicated that none of the analyzed "white" pomegranate tissues are able to synthesize mRNA corresponding to the *PgLDOX* gene (leucoanthocyanidin dioxygenase, also called *ANS*, anthocyanidin synthase), which is one of the central structural genes in the anthocyanin-biosynthesis pathway. HPLC analysis revealed that none of the "white" pomegranate tissues accumulate anthocyanins, whereas other flavonoids, corresponding to biochemical reactions upstream of LDOX, were present. Molecular analysis of the "white" pomegranate revealed the presence of an insertion and an SNP within the coding region of *PgLDOX*. It was found that the SNP does not change amino acid sequence and is not fully linked with the "white" phenotype in all pomegranate accessions from the collection. On the other hand, genotyping of pomegranate accessions from the collection and segregating populations for the "white" phenotype demonstrated its complete linkage with the insertion, inherited as a recessive single-gene trait. Taken together, the results indicate that the insertion in *PgLDOX* is responsible for the "white" anthocyanin-less phenotype. These data provide the first direct molecular, genetic and chemical evidence for the effect of a natural modification in the *LDOX* gene on color accumulation in a fruit-bearing woody perennial deciduous tree. This modification can be further utilized to elucidate the physiological role of anthocyanins in protecting the tree organs from harmful environmental conditions, such as temperature and UV radiation.

## Introduction

Pomegranate (*Punica granatum* L.) is known for its appealing colors, with high color variability reported among pomegranate accessions from different collections around the world, including Iran [1], China [2], Turkey [3], Israel [4], Turkmenistan [5] and the USA [6]. This variability is displayed mostly in the fruit skin, but also in the arils and flowers. Pomegranate fruit skin displays a wide array of colors ranging from white-yellow, green, pink, and red to dark purple. Pomegranate aril color varies from white to deep red, and the flower colors vary from white-yellow and orange-red to deep red. Young leaves tend to have a reddish color and turn green when the leaf matures, with no marked variability among accessions for this characteristic [7].

Anthocyanins, which belong to the flavonoid family, are the major pigments responsible for pomegranate fruit color. Six major anthocyanin compounds have been identified in pomegranate fruit, including mono- and diglucosides of cyanidin (red), pelargonidin (orange) and delphinidin (purple) [8–11]. The color variability in various tissues and among different pomegranate accessions stems from different contents of anthocyanin and different relative amounts of its derivatives [8]. Other flavonoids and polyphenols might also contribute some of the yellowish colors seen in pomegranate fruits [12].

Anthocyanins are commercially important molecules due to their health-beneficial properties and attractive colors. The potential health benefits of plant-derived anthocyanin-rich foods have been reviewed, with a focus on the role of anthocyanins in cancer prevention [13], cardiovascular disease prevention [14], antimicrobial and anti-inflammatory effects [15–16], obesity control, diabetes control, and improvement of visual and brain functions [17–19]. Anthocyanins also contribute to the fruit's color, which significantly affects market success. In addition, anthocyanins play an important role in protecting plant tissues against UV irradiation [20–21] and photoinhibition [22]. These pigments have also been shown to double the shelf life of tomatoes by delaying over-ripening and reducing susceptibility to gray mold [23–24].

**Fig 1:**
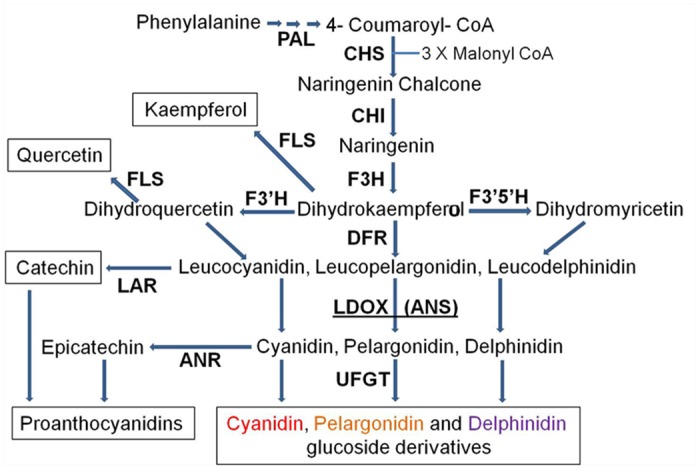
Schematic representation of the flavonoid-biosynthesis pathway leading to the production of anthocyanins and proanthocyanidins. Enzyme name abbreviations are as follows: PAL, phenylalanine ammonia-lyase; CHS, chalcone synthase; CHI, chalcone isomerase; F3H, flavanone 3-hydroxylase; F3'H, flavonoid 3'-hydroxylase; F3'5'H, flavonoid 3',5'-hydroxylase; FLS, flavonol synthase; DFR, dihydroflavonol reductase; LDOX, leucoanthocyanidin oxidase; ANS, anthocyanidin synthase; LAR, leucoanthocyanidin reductase; ANR, anthocyanidin reductase; UFGT, UDP glucose:flavonoid 3-O-glucosyltransferase.

The genetic control of anthocyanin biosynthesis is relatively well understood in many plant species, including annuals and woody perennials. The main structural genes involved in anthocyanin biosynthesis include: phenylalanine ammonia lyase (PAL), chalcone synthase (CHS), chalcone isomerase (CHI), flavanone 3-hydroxylase (F3H), dihydroflavonol 4-reductase (DFR), leucoanthocyanidin dioxygenase (LDOX), and UDP glucose:flavonoid 3-O-glucosyltransferase (UFGT) [25–26] (Fig 1). In addition, regulatory genes control the temporal and spatial expression of the structural genes. The main regulatory genes encode proteins containing a R2R3-MYB domain, a basic helix—loop—helix (bHLH) domain and WD40 repeats. Combinations of the R2R3-MYB, bHLH and WD40 proteins and their interactions determine the set of genes to be expressed [27–29].

The structural and regulatory genes involved in the anthocyanin-biosynthesis pathway are highly conserved among different plant species, including pomegranate, in which three regulatory genes have been described: *PgWD40*, *PgAn1* (bHLH) and *PgAn2* (MYB) [30]. Activity of the pomegranate regulatory gene *PgWD40* was demonstrated in an arabidopsis *ttg1-9* mutant and was shown to be dependent on MYB function. In addition, high correlations have been found between the expression levels of the three regulatory genes and those of two key biosynthetic genes of the anthocyanin pathway: *PgDFR* and *PgLDOX*. The expression of *PgWD40* and *PgAn2* (MYB) genes is also highly correlated with cyanidin (red pigment) accumulation in the pomegranate fruit skin [30].

Mutations in anthocyanin-biosynthesis genes that alter color production are highly important as they contribute to our understanding of the genetic and molecular mechanisms controlling anthocyanin accumulation in pomegranate. Furthermore, new phenotypes can be employed as cultivars with beneficial traits. One such mutation is the "white" pomegranate that is characterized by the absence of the typical pomegranate color in plant tissues including flowers, fruit (skin and arils) and leaves.

White pomegranates have been reported from China [31], India [32] and Iran [33–34]. However, the term “white” in those reports seemed to relate mostly to non-red pomegranates, and it is not clear whether “white” reflected partial or total absence of anthocyanins in the plant tissues, or which tissues were affected. The genetic nature of the "white" pomegranate, as specified in the present manuscript, has not yet been determined. In this study, molecular and genetic approaches were combined with chemical analysis to characterize and identify the nature of a "white" pomegranate accession. The "white" phenotype was initially characterized by the absence of any sign of anthocyanins in the leaves, fruit (skin and arils) or flowers. Our analyses indicate that the cause for the "white" phenotype is a mutation in *PgLDOX*, a structural gene involved in the anthocyanin-biosynthesis pathway in pomegranate.

## Materials and Methods

### Plant material

"White" pomegranate accession P.G.254-265 and the red cv. Wonderful (P.G.100-1) were from the live pomegranate tree collection at the Newe-Ya’ar Research Center located in northern Israel (S1 Fig). Samples from various tissues were collected from spring (end of April) to autumn (end of September) 2010. The first developmental stage was collected from unfertilized hermaphroditic flowers, at the end of April. Later, developing fruits were collected at successive one-month intervals, until the end of September. Overall, six different stages were collected: flower (stage 1), young fruit (stages 3 and 6), nearly mature fruit (stage 8), ripened fruit (stage 10), and over-ripened fruit according to commercial practices (stage 12). Four to eight flowers, fruits from each sampling stage and young leaves (up to three weeks from bud burst) were collected. Flower samples were taken only from the sepals, and the fruit samples were separated into skin and arils. Collected tissues were stored at -80°C for further analysis.

### Reversed phase HPLC analysis of anthocyanins and other flavonoids

Tissue samples were homogenized in 80% methanol supplemented with 2 mmol L^-1^ NaF (1:2, w/v) using a cold mortar and pestle. The suspension was centrifuged (10,000 rpm for 10 min at 4°C, Sorvall Instruments RC5C, rotor no. SS-34), and the supernatant was filtered through a 0.45-°m PTFE (polytetrafluoroethylene) filter before injection.

Flavonoid composition was analyzed by reversed phase HPLC following Borochov-Neori *et al*. [10] with a LaChrom Merck Hitachi HPLC system consisting of Pump L-7100, column oven L-7350, mixer-degasser L-7614 and Rheodyne manual injector, coupled with a diode array detector with 3D feature (Multiwavelength Detector, Jasco MD-2010 Plus), interface (Jasco LC-Net II/ADC) and scientific software (EZChrom *Elite* Client/Server version 3.1.6 build 3.1.6.2433). A column of LiChrospher 100 RP-18 (5-°m particle size in 250 x 4 mm LichroCART cartridge, Merck Millipore) with a guard column (LiChrospher 100 RP-18, 5-°m particle size in 4 x 4 mm LichroCART cartridge) was used.

The binary mobile phase consisted of phosphoric acid (0.1% v/v, pH 2.4) (solution A) and acetonitrile (solution B). Elution was carried out with the following gradient scheme: 10% solution B at 1 ml min^-1^, 0–10 min; 10–20% solution B at 1 ml min^-1^, 10–15 min; 20% solution B at 1–0.6 ml min^-1^ (Flow rate is changing during this period from 1 to 0.6 ml min^-1^), 15–16 min, and 20% solution B at 0.6 ml min^-1^, 16–26 min. Following anthocyanin elution, the column was washed with 80% solution B at 1 ml min^-1^ for 10 min and equilibrated with 10% solution B at 1 ml min^-1^ for an additional 10 min. Acetonitrile was HPLC-grade (LiChrosolv, Merck). Column-filtered water was further distilled by Corning Megapure System MP-6A, and passed through a 0.20-°m nylon membrane. Phosphoric acid and NaF were of analytical grade.

A flavonoid-standard library was constructed from delphinidin 3,5-diglucoside, cyanidin 3,5-diglucoside, pelargonidin 3,5-diglucoside (Apin Chemicals, Limited (UK)), delphinidin 3-glucoside, cyanidin 3-glucoside, pelargonidin 3-glucoside (Polyphenols Laboratories AS), catechin, epicatechin (Sigma-Aldrich Co., Rehovot, Israel), kaempferol 3-glucoside and quercetin 3-β-glucoside (Fluka Analytical, Sigma-Aldrich Co., Rehovot, Israel). Flavonoid identification was performed by the software on the basis of UV/VIS absorbance spectra and retention times. Peak area was also calculated by the software. A detection limit (minimal peak area) of 15,000 and 100,000 was set for anthocyanins and other flavonoids, respectively. Under the conditions employed in this study, the chromatogram peak areas were linearly correlated with the concentrations of the corresponding phenolic compounds.

### Nucleic acid isolation and cDNA synthesis

Total RNA was extracted from young leaves, flowers, fruit skin and arils of the pomegranate as described by Meisel *et al*. [35] for plants containing high amounts of polysaccharides and polyphenolic compounds. Tissues from at least three flowers, fruits or leaves were combined and then extracted. Each total RNA sample was incubated with 1 MBU RNase-free DNase *I* (Epicentre Biotechnologies, Madison, WI, USA) for 30 min at 37°C to remove co-extracted genomic DNA. Total RNA was quantified by spectrophotometry (ND-1000 spectrophotometer, NanoDrop Technologies, Wilmington, DE, USA), and the quality was assessed by the ratio of absorbances at 260 and 280 nm (A_260_/A_280_) and A_260_/A_230_ (1.8 to 2.2 and ≥1.8, respectively, indicating good quality), and by the demonstration of intact rRNA bands following electrophoresis on an agarose gel. After DNase treatment, first-strand cDNA was synthesized from 1.0 μg of total RNA using Verso reverse transcriptase and oligo (dT) by Verso cDNA kit (Thermo Scientific, Epson, UK), according to the manufacturer's instructions. First-strand cDNA synthesis was carried out in triplicate for each sample (to minimize variation in RNA template levels) and then samples were pooled.

Genomic DNA was extracted from fresh young leaves using the hexadecyl trimethyl ammonium bromide—polyvinyl pyrrolidone (CTAB-PVP) method [36]. DNA concentrations were quantified by agarose gel electrophoresis and spectrophotometrically (NanoDrop).

### Semi-quantitative RT-PCR

The expression levels of three regulatory genes and eight structural genes predicted to be involved in pomegranate anthocyanin biosynthesis were analyzed by semi-quantitative RT-PCR. The genes were isolated from pomegranate fruit skin based on their homology to genes known to be involved in anthocyanin pathways in other plants. The sequences of *PgDFR*, *PgAn2* (MYB), *PgAn1* (bHLH) and *PgWD40* were taken from Ben-Simhon *et al*. [30]. The sequences of *PgPAL-4*, *PgCHS-3*, *PgCHI*, *PgF3H-2*, *PgF3’H*, *PgF3’5’H* and *PgLDOX* were derived from the pomegranate transcriptome recently established by our group [37].

The pomegranate-specific primers used for amplification of the anthocyanin-biosynthesis genes and their corresponding PCR product lengths are listed in S1 Table. PCR conditions were as follows: 95°C for 4 min, followed by 28–31 cycles of 95°C (30 s), 50°C–60°C (30 s) and 72°C (60 s), and a final extension at 72°C for 7 min and 4°C for 30 min. To ensure that amplifications were within the linear range, PCRs were performed with 27, 30, 33, and 40 cycles. The correct PCR conditions were validated by obtaining serial dilutions of the cDNA samples and then determining their band densities on 1% agarose gels.

### Quantitative RT-PCR analysis

qRT-PCR was performed using the StepOne Plus system (Applied Biosystems) and the Fast SYBR Green Master Mix (Applied Biosystems). Reactions were performed in triplicate using 5 μL of Master Mix, 0.2 μM of each primer, 1 μL of diluted cDNA and nuclease-free water to a final volume of 10 μL. The pomegranate-specific primers used for the qRT-PCR analysis are listed in S2 Table. Thermocycling conditions were set as an initial polymerase activation step for 20 sec at 95°C, followed by 40 cycles of 3 sec at 95°C for template denaturation and 30 sec at 60°C for annealing/ extension step. Fluorescence was measured at the end of each annealing step. Afterwards, a dissociation protocol with a gradient from 60°C to 95°C was used for each primer pair to verify the specificity of the qRT-PCR reaction and the absence of primer dimer. The raw data were analyzed with the StepOne software, version 2.2.1. Expression was normalized to pomegranate Ribosomal Protein S, named *PgRPSΙΙ*, derived from the pomegranate transcriptome [37]. *PgRPSII* was selected for normalization due to its consistent transcript level different fruit tissues and different pomegranate accessions, with crossing threshold (Ct) values changing by < 2. The sample from developmental stage 3 was used as a calibrator with a nominal value of 1.Error bars shown in qRT-PCR data are technical replicates, representing the means _ SE of three replicate qRT-PCR reactions.

### Sequencing of *PgLDOX*

The sequence of *PgLDOX* was derived from 454 sequencing analysis of RNA extracted from several pomegranate accessions used to establish the pomegranate transcriptome [37] and from partial genomic- sequencing by Illumina which was done on DNA from a pomegranate accession which is producing anthocyanin (unpublished data). PCR products were purified and cleaned before sequencing by Illustra ExoStar One-Step kit (GE Healthcare Life Sciences, Little Chalfont, UK). Cycle sequencing reactions were performed with the BigDye cycle sequencing kit (Applied Biosystems, Foster City, CA, USA) and were analyzed using capillary separation on an ABI3130x Genetic Analyzer (Applied Biosystems). Mulatlin software [38] was used to compare *PgLDOX* sequences from different pomegranate accessions. BLAST program [39] was used to search for PgLDOX homologs in the NCBI database. Multiple sequence alignment of the deduced amino acid sequences of PgLDOX and LDOX proteins from other plants was constructed using Clustal Omega software and a phylogenetic tree was constructed using Clustal W2-Phylogeny software.

Two polymorphic sites were identified in *PgLDOX*: a single-nucleotide polymorphism (SNP) C/T at position 1,008 downstream of the ATG initiation codon, and an insertion. The SNP was identified by sequencing the *PgLDOX* gene in 20 different accessions from the pomegranate collection that displayed "white" or colored phenotypes. The pomegranate-specific primers used for amplification and sequencing the SNP site are listed in S2 Table.

The insertion was detected in two steps. (1) PCR analysis using primers flanking the insertion (primer list in S2 Table). It was done under the following conditions: 95°C for 4 min, followed by 35 cycles of 95°C (30 s), 55°C (30 s) and 72°C (60 s), and a final extension at 72°C for 7 min and 4°C for 30 min, using Taq RED Master Mix Kit (Apex, Genesee Scientific, San Diego, CA, USA). These PCR conditions could not amplify the insert, and therefore no PCR product was obtained from accessions containing the insert sequence. (2) Amplification of the insert to determine its sequence. We used the AFLP method [40]. Essentially, genomic DNA of "white" and red cultivars was digested with *MseI*. The DNA digest was ligated to MseI adaptor and the ligation products were amplified by a primer based on the MseI adaptor and a PgLDOX-R7 primer from the *PgLDOX* gene. PCR conditions were as follows: 95°C for 4 min, followed by 30 cycles of 95°C (30 s), 55°C–60°C (30 s) and 72°C (60 s), and a final extension at 72°C for 10 min and 4°C for 30 min. Re-amplification of the PCR products was done with nested primers R5 and R2 (primer list in S2 Table). After the third re-amplification, a 150 bp and 250bp fragments of DNA from the "White" and red accessions respectively, were separated on 1% agarose gel. The bands were isolated, purified and sequenced as described previously.

### Establishment of a genetic population and its genotyping

An F1 population was constructed by crossing the colored accession 'Nana1' (P.G.149-50) as the female parent with the "white" pomegranate (P.G.164-65) as the male parent. Randomly selected F1 progeny were self-pollinated to generate five F2 populations with a total of 464 progeny. One F2 population of 91 plants was selected for genotyping for the two markers identified in *PgLDOX*: the SNP and the insertion. SNP genotyping was performed by high-resolution melting (HRM) technique [41–42] in an Eco real-time PCR system (Illumina). High-quality genomic DNA was assessed by NanoDrop (A_260_/A_280_ and A_260_/A_230_), and an equal amount of genomic DNA (100 ng°l^-1^) was determined in all samples. PCR primers for the *PgLDOX* HRM marker are generating a 116-bp product and listed in S2 Table. For the HRM program, Fast SYBR Green Master Mix (Applied Biosystems) was used according to the manufacturer's instructions. The unknown *PgLDOX* sequences from the progeny and the known *PgLDOX* sequences from the parents were compared to determine the genotype of each progeny.

The insertion marker was genotyped in the F2 population by PCR analysis using the primers flanking the insertion under the same conditions mentioned in Sequencing of *PgLDOX* section. To amplify a partial sequence from the insert we used a primer based on the 18bp that were sequencing by the AFLP method (primer list in S2 Table).

## Results

### Phenotypic characteristics of the "white" pomegranate

**Fig 2:**
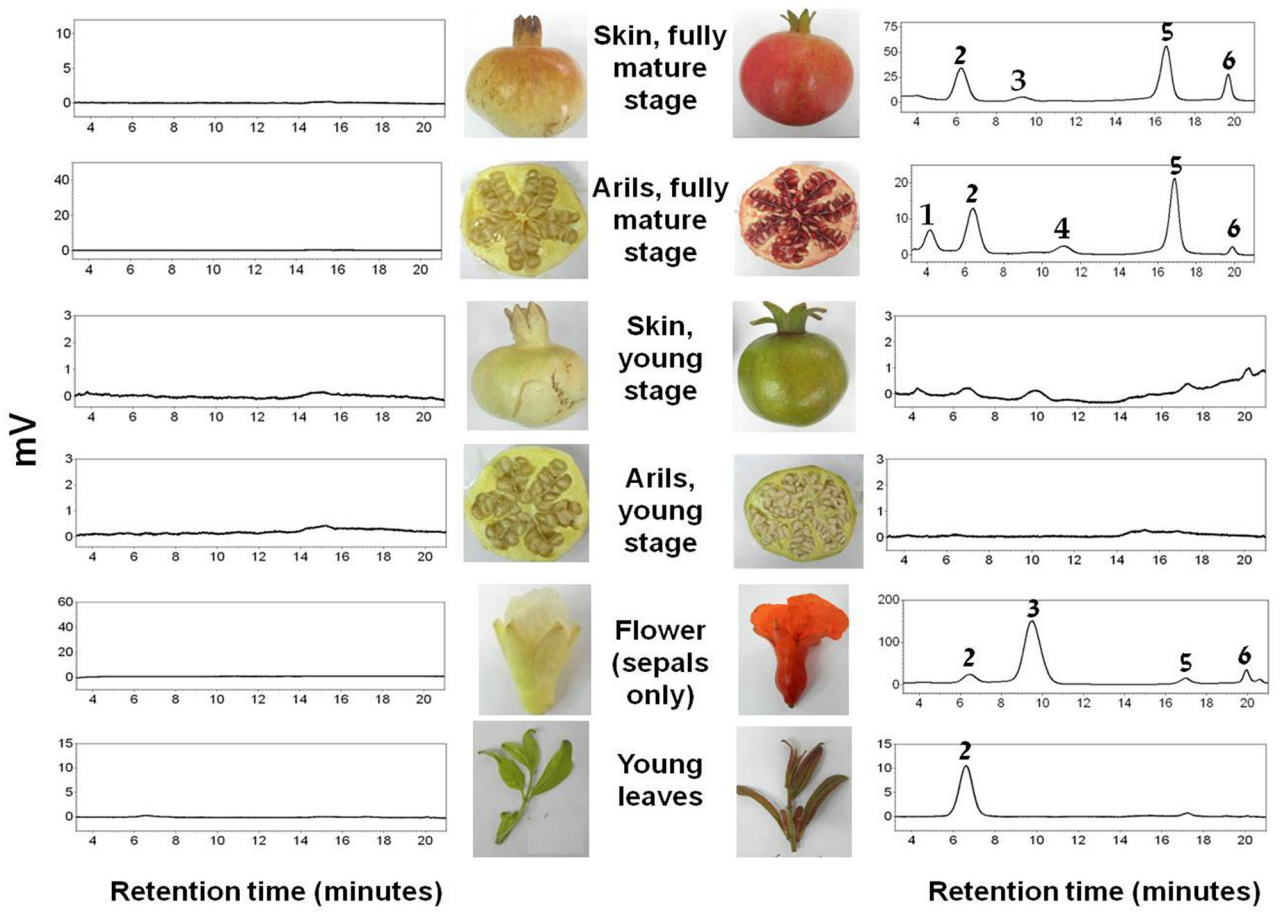
HPLC chromatograms at 520 nm of methanolic extracts of different tissues of cv. Wonderful (right) and the "white" pomegranate (left). Chromatogram peak identities were based on the UV/Vis spectrum and retention times: (1) delphinidin 3,5-diglucoside; (2) cyanidin 3,5-diglucoside; (3) pelargonidin 3,5-diglucoside; (4) delphinidin 3-glucoside; (5) cyanidin 3-glucoside; (6) pelargonidin 3-glucoside.

Flower petals and sepals of the "white" pomegranate are yellow-white (Fig 2). The skin color at the different developmental stages of the fruit (stages 3–10) varies from green to yellowish, and the aril color during these same developmental stages is white (S1A Fig). Fully mature fruit (stage 12) has whitish arils and skin with a brownish blush, typical of mild sunburn damage (Fig 2). A tiny red spot appears on the arils and skin of over-ripened fruit left on the tree during the winter season. The young leaves of the "white" pomegranate are green with no traces of red color, in contrast to their strong red color in most pomegranate accessions (Fig 2). Since the "white" pomegranate contains pigments such as chlorophyll, minute amounts of carotenoids and some flavonoids (data not shown), the term "white" was chosen, rather than albino or colorless pomegranate.

### HPLC analysis of anthocyanin content in the "white" pomegranate

The phenotypic characteristics of the "white" pomegranate strongly suggested that all of its tissues lack anthocyanins. To confirm this, HPLC analysis was conducted using the red cv. Wonderful as a control on young leaves, sepals, arils and skin of young fruit (stage 8) and fully mature fruit (stage 12).

The results in Fig 2 and Table 1 indicate that the red cv. Wonderful contained anthocyanins in most of the analyzed tissues, whereas the "white" pomegranate lacked these pigments in all of the analyzed tissues. Mono- and diglycoside derivatives of cyanidin (red pigment), delphinidin (purple pigment) and pelargonidin (orange pigment) were detected in the red cv. Wonderful, their levels varied among the different tissues. The leaves contained only cyanidin 3,5-diglucoside. The sepals contained mostly pelargonidin derivatives and small amounts of cyanidin. The skin of the fully mature fruit contained both cyanidin and pelargonidin derivatives while the arils of the fully mature fruit contained cyanidin, pelargonidin and also delphinidin derivatives. In addition, no anthocyanins were observed in the arils and skin of the fruit at the young green stage. In contrast to cv. Wonderful, no anthocyanins were detected in any of the tissues originating from the "white" pomegranate accession. This result corroborates the visual observations and suggests that the "white" pomegranate does not produce anthocyanins in any of the examined tissues.

**Table 1:** Comparison of anthocyanin composition and quantity in different tissues of the "white" pomegranate and of the red cv. Wonderful.

Tissue	Anthocyanin	Chromatogram peak area (peak area/mg tissue)	
		cv. Wonderful	"white" pomegranate
**Young leaves**	Cyanidin 3,5-diglucoside	488,383	ND
**Flower (sepals only)**	Cyanidin 3,5-diglucoside	85,825	ND
	Pelargonidin 3,5-diglucoside	903,953	ND
	Cyanidin 3-glucoside	53,639	ND
	Pelargonidin 3-glucoside	55,552	ND
**Arils (mature stage)**	Delphinidin 3,5-diglucoside	13,977	ND
	Cyanidin 3,5-diglucoside	38,740	ND
	Delphinidin 3-glucoside	7,563	ND
	Cyanidin 3-glucoside	58,650	ND
	Pelargonidin 3-glucoside	3,368	ND
**Skin (mature stage)**	Cyanidin 3,5-diglucoside	239,298	ND
	Pelargonidin 3,5-diglucoside	37,906	ND
	Cyanidin 3-glucoside	352,831	ND
	Pelargonidin 3-glucoside	99,508	ND

ND, not detected.

### Expression of key biosynthetic and regulatory genes of the anthocyanin pathway in the "white" pomegranate

**Fig 3:**
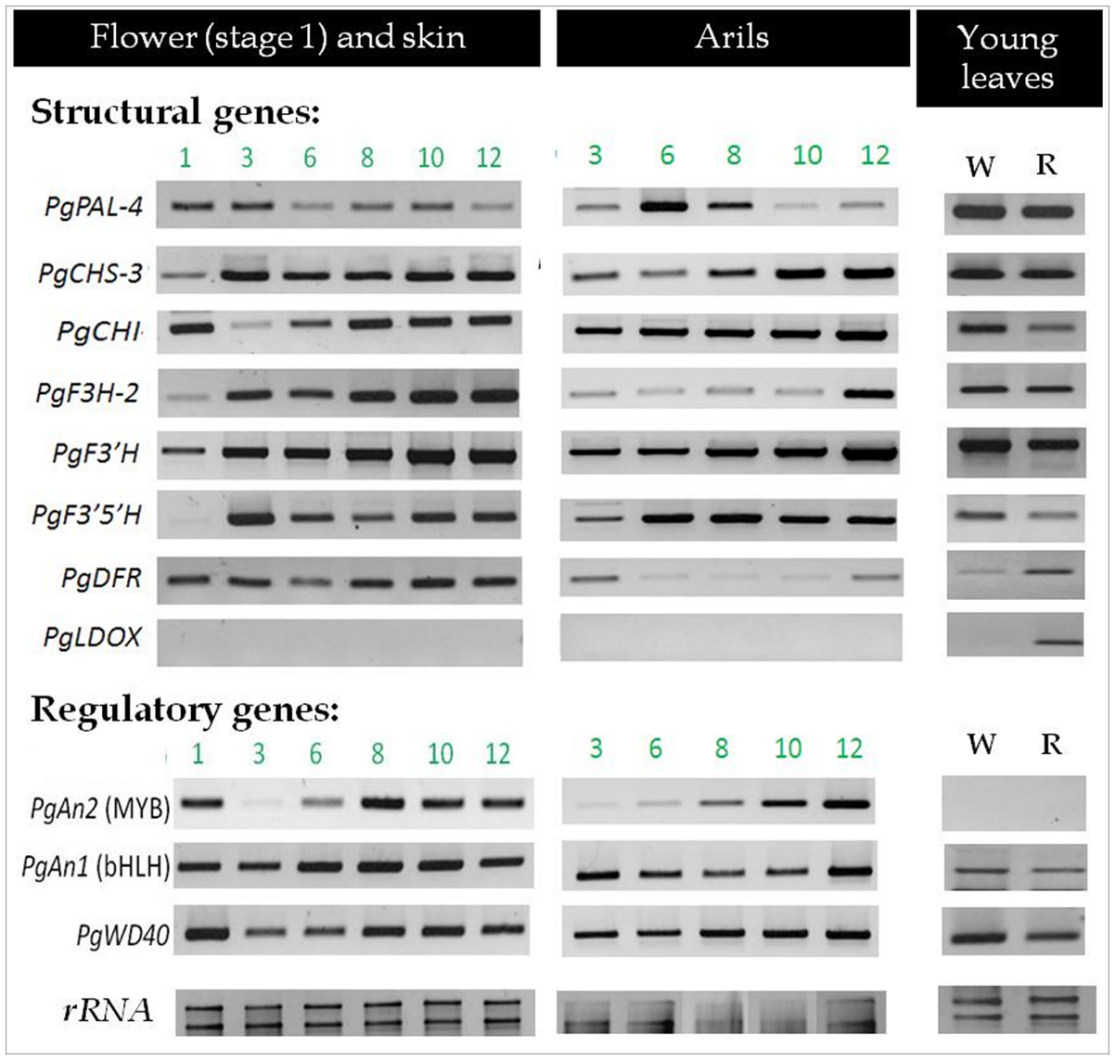
Expression patterns of the studied anthocyanin biosynthesis structural and regulatory genes in different tissues of the "white" pomegranate. The expression patterns of the structural and regulatory genes that are predicted to be involved in anthocyanin biosynthesis in pomegranate, are represented. The analysis was done on skin and arils of the "white" pomegranate, during fruit development [from flowers (stage 1) to fully mature fruit (stage 12)] and on young leaves. W, young leaves of the "white" pomegranate; R, young leaves of the red cv. Wonderful. Semi-quantitative RT-PCR analysis was performed as described in Materials and methods. Samples were normalized to 18S and 28S ribosomal RNA (*rRNA)* as the reference gene for constitutive expression. PCR products were separated on a 1% agarose gel.

To identify and understand the molecular mechanism responsible for the lack of anthocyanin in the "white" pomegranate, the expression levels of the following genes, involved in anthocyanin biosynthesis in pomegranate, were analyzed: structural genes *PgPAL-4*, *PgCHS-3*, *PgCHI*, *PgF3H-2*, *PgF3’H*, *PgF3’5’H*, *PgDFR*, and *PgLDOX*, and regulatory genes *PgAn2* (MYB), *PgAn1* (bHLH) and *PgWD40*. Semi-quantitative RT-PCR was conducted on samples from skin and arils of fruit at different developmental stages and from young leaves (Fig 3). Alignment of the deduced amino acid from pomegranate genes and their corresponding sequences in other plant gene homologues is presented in S2 Fig.

**Fig 4:**
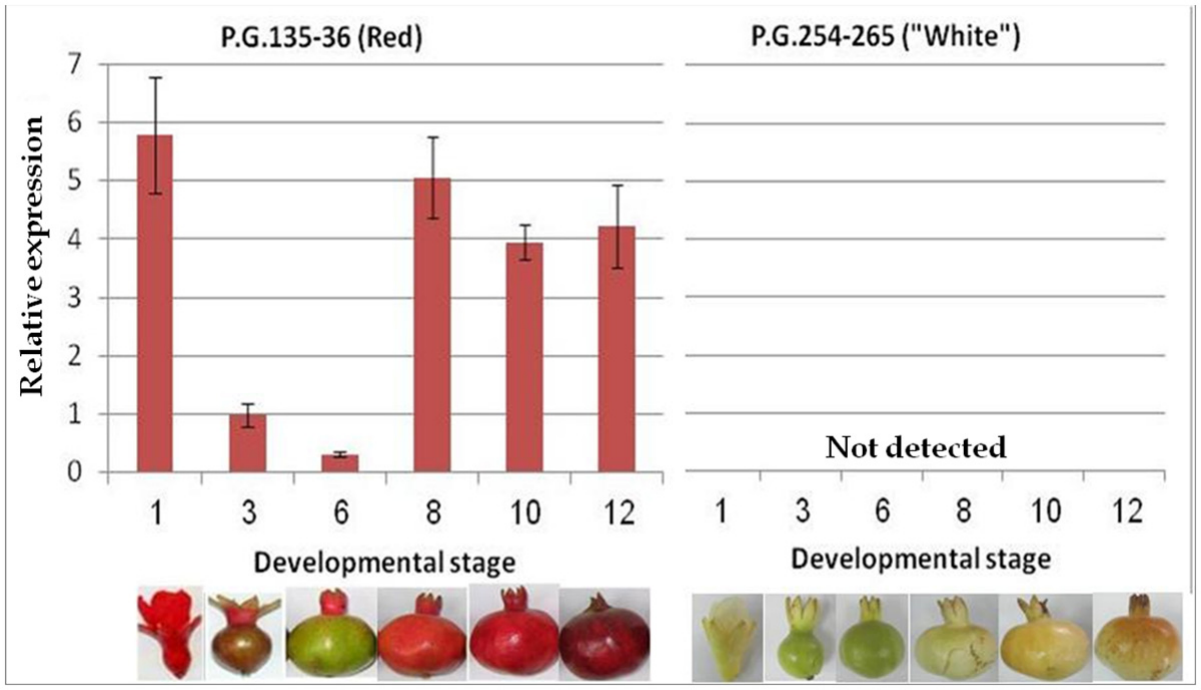
Expression of *PgLDOX* in red and “White” pomegranate during fruit developmental stages. Quantitative RT-PCR analysis was performed on samples from red (left) and “white” (right) pomegranate fruit skin during different developmental stages ranging from flower (stage 1) to fully mature fruit (stage 12). The relative expression is expressed as the fold increases relative to stage 3. Error bars are SE for three replicate reactions. The various fruit developmental stage of the red and “white” pomegranate are displayed in the bottom of the figure.

All of the structural and regulatory genes involved in anthocyanin biosynthesis in pomegranate, except for *PgLDOX*, were expressed in all of the examined tissues of the "white" pomegranate. These genes have a characteristic expression pattern with a tendency to increase with fruit maturation. *PgLDOX* is the only gene that was not expressed in any of the tissues of the "white" pomegranate, i.e. flower, skin and arils at different fruit developmental stages and young leaves. In our previous work we measured the expression of *PgLDOX* in two red cultivars: cv. Wonderful and P.G.135-36, by semi-quantitative analysis. High and positive correlation was found between the expression level of *PgLDOX*, and between the amount of cyanidin (red pigment) and the phenotype observed [30]. To verify these results we used quantitative RT-PCR analysis to measure the expression of *PgLDOX* in the skin of red cultivar P.G. 135–36 and the "White" pomegranate (Fig 4). Expression of *PgLDOX* in the red cultivar was characteristic to what was found for *PgLDOX* measured by semi-quantitative analysis. In addition, no expression was detected in any of the developmental stages in the skin of the "white" pomegranate, as indicated by qRT-PCR analysis.

Expression of the transcription factor *PgAN2* (MYB) was also not detected in the young leaves of the "white" pomegranate. However, it was also not detected in young leaves of the red cv. Wonderful. Thus, the expression of *PgAN2* (MYB) seemed to be tissue-specific and not related to the "white" phenotype.

### Comparison of flavonoid and proanthocyanidin contents in mature fruit skin of the "white" pomegranate and red cv. Wonderful

The indication that the "white" phenotype may result from null expression of the *PgLDOX* gene predicted that flavonoid intermediates upstream of the LDOX function would accumulate, whereas components which are downstream of the LDOX stage would be absent in the "white" pomegranate (as noted for anthocyanins). Following this logic, HPLC analysis was conducted to compare the accumulation of various flavonoids in the skin of mature fruit (stage 12) of the "white" pomegranate and the red cv. Wonderful.

**Fig 5:**
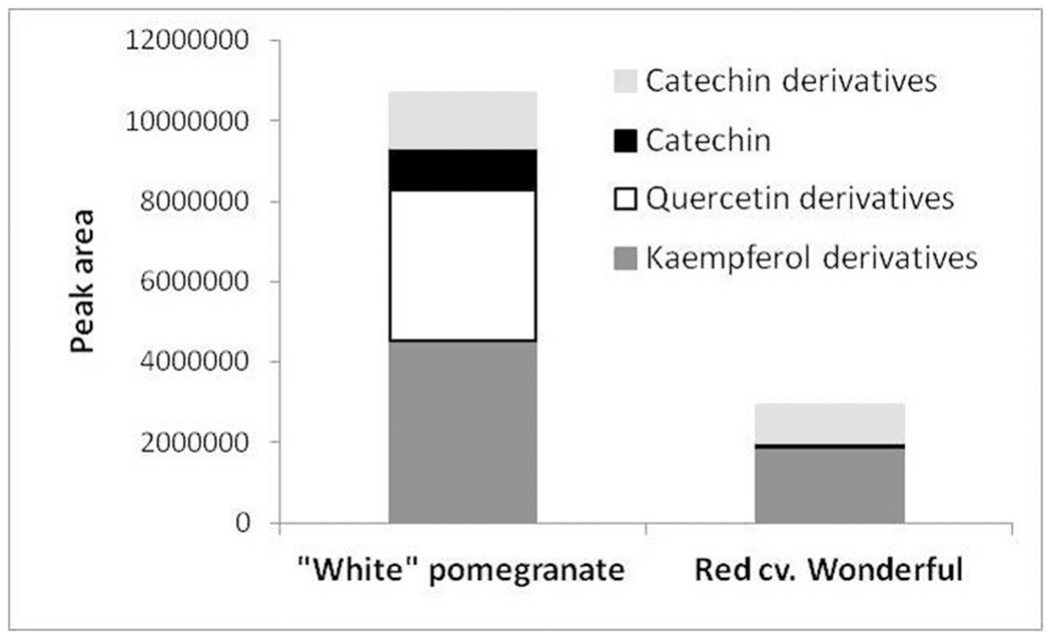
Comparison of the content of flavonoid and proanthocyanidin precursors in the skin of mature fruit of the "white" pomegranate and the red cv. Wonderful.

Although no anthocyanins were detected in any of the "white" pomegranate tissues (Fig 2), flavonoids corresponding to the upstream stages of LDOX function accumulated in the fruit skin of this accession. Thus, kaempferol derivatives (intermediates of the pelargonidin orange pigment), quercetin derivatives (intermediates of the cyanidin red pigment), catechin and catechin derivatives (intermediates of the proanthocyanin pigment) accumulated in the "white" pomegranate in significantly higher amounts than in the red cv. Wonderful (Fig 5). These data strongly suggest that the flavonoid pathway is blocked at the LDOX stage in the "white" pomegranate (see also Fig 1).

### Comparison of genomic sequences of *PgLDOX* in "white" and colored pomegranate accessions

The lack of expression of *PgLDOX* in all "white" pomegranate tissues suggested that the inability to produce anthocyanin in the tissues of this accession might be due to a mutation that affects *PgLDOX* expression. This mutation could occur within the gene itself, in the gene’s flanking regions or in other regulatory or structural genes that affect *PgLDOX* expression. To examine these possibilities, the genomic sequences of *PgLDOX* in the "white" pomegranate were compared with those in other colored pomegranate accessions from the Newe Ya'ar collection.

**Fig 6:**
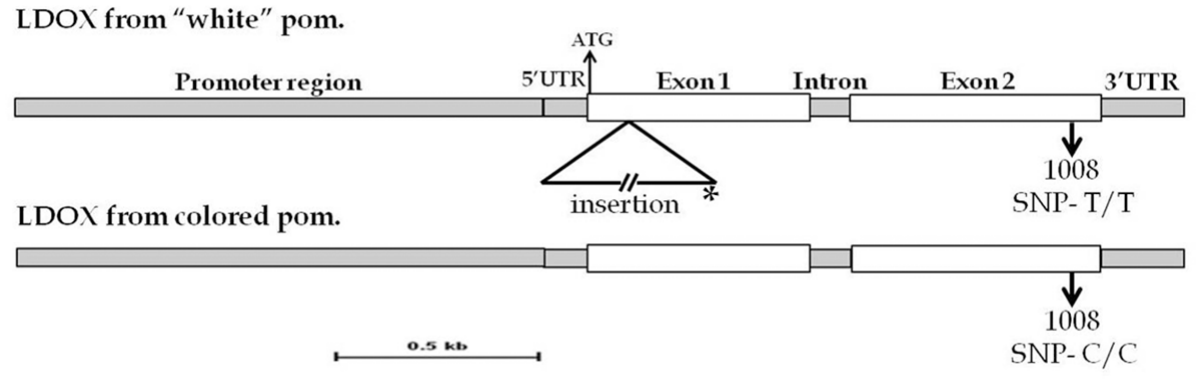
Comparison of genomic sequences of PgLDOX in the "white" and colored pomegranate accessions. Schematic representation of *PgLDOX* genomic structure from the "white" and colored pomegranates. The white boxes indicate exons and the gray boxes indicate translated regions. The insertion in *PgLDOX* from the "white" pomegranate is shown as a triangle and located between positions 90–91 downstream of the ATG initiation codon. The SNP is located 1,008 bp downstream of ATG and does not change the amino acid sequence. [*] Represents the site of the short sequence from the insertion that was amplified and sequenced.

The sequence of *PgLDOX* gene was determined by combination of two data source: first, 454 sequencing analysis of RNA extracted from four different colored pomegranate accessions, used to establish the pomegranate transcriptome [37] and second, partial sequencing of genomic DNA obtained by Illumina analysis (unpublished data). The transcriptome data suggested the presence of two LDOX contigs: pom_rep_c1451 (TSA accession number GBGR01001440) and pom_rep_c28575 (TSA accession number GBGR01028523). RT-PCR analysis using specific primers for each of the contigs indicated that the main expressed contig is pom_rep_c1451, since contig pom_rep_c28575 was not found to be transcribed in different tissues and pomegranate cultivars, and probable is some pseudo-gene (data not shown). The length of contig pom_rep_c1451 is 1,367 bp. In addition, two non- overlapping contigs: GenBank JI988744.1 and JI989504.1, annotated as *LDOX* were also found by Ono *et al*. [43] in pomegranate. RT-PCR with specific primers for each of these contigs revealed that only one contig which is identical to the 3'-end part of pom_rep_c1451 was transcribed (data not shown). Since there is partial genomic sequence data for pomegranate, it is difficult to assess how many copies of *PgLDOX* are in pomegranate. However, based on the transcriptome data, RT-PCR expression analysis and genetic analysis, we argue that *PgLDOX* (TSA accession number GBGR01001440) encodes the major LDOX in pomegranate. In addition to the transcriptome data, the genomic data enabled to add 1,223 bp in the promoter region and to discover a single intron between nucleotide positions 505–506 within the coding region, with a length of 94 bp (Fig 6). Therefore, the total genomic region of *PgLDOX* gene that was sequenced is 2,684 bp.

Within the full-length sequence, an open reading frame (ORF) of 1,071 bp was found, predicting a protein of 357 amino acid residues. Comparison of the deduced PgLDOX protein sequence to those in the GenBank database, revealed highest sequence similarity with LDOX from *Eucalyptus* (85% amino acid identity), grape and a *Cyclamen graecum* (83% amino acid identity), and with fruit tree species that belong to the family Rosaceae, including peach, apple and strawberry (80%–82% amino acid identity). Lower sequence similarity (77%–80% amino acid identity) was found with LDOX isolated from species of the families Solanaceae and Brassicaceae, including LDOX from *Arabidopsis thaliana* (79%). A phylogenetic tree based on the various LDOX gene homologs from the pomegranate and other plant species is presented in S3 Fig. According to this tree, PgLDOX clustered together with LDOX from *Eucalyptus* to form a distinct clade of LDOX proteins.

Comparison of the genomic DNA sequences of *PgLDOX* from the "white" and 20 colored pomegranate accessions revealed that this gene is well-conserved among different pomegranate accessions, except for one SNP in the ORF of *PgLDOX*. This SNP is located in position 1,008 downstream of the ATG initiation codon. Two alleles for this SNP were found: the "white" pomegranate contained a T/T combination and the colored accessions contained C/C, except for one accession which was heterozygous for this SNP (Fig 6 and Table 2). However, it was also found that colored siblings of the "white" pomegranate shared the same alleles as the "white" siblings (Table 2). In addition, this SNP (C to T) did not affect the deduced protein sequence. For this reason, it was assumed that this SNP is not responsible for the "white" phenotype.

**Table 2:** Fruit-color characteristics and allelic genotypes of *PgLDOX* in different pomegranate accessions grown in the Newe Ya’ar pomegranate collection.

Accession number	Common name	Fruit skin color	Aril color	SNP	Insertion in both alleles of *PgLDOX*
P.G.254-265	6/35(3)	"White"	White	T/T	+
P.G.164-65	6/41(3)	"White"	White	T/T	+
-	6/24(3)	Pink-yellow	White	T/T	-
P.G.255-266	6/28(3)	Dark pink-yellow	White	T/T	-
P.G.170-71	6/32(3)	Pink-yellow	White	T/T	-
P.G.100-1	Wonderful	Red	Dark red	C/C	-
P.G.120-21	Rosh Hapered	Dark pink	Pink	C/C	-
P.G.127-28	Black	Black	Pink-red	C/C	-
P.G.128-29	Acco	Red	Dark red	C/C	-
P.G.218-229	Emek	Red	Dark red	C/C	-
P.G.130-31	Shani-Yonay	Red	Dark red	C/T	-
P.G.134-35	Kamel	Bordeaux	Red	C/C	-
P.G.149-50	Nana1	Dark pink-yellow	Light red	C/C	-
P.G.160-61	Moti	Red-yellow	Light pink	C/C	-
P.G.201-212	ERS	Dark pink	White	C/C	-
P.G.204-215	C13	Pink-yellow	Light pink	C/C	-
P.G.213-224	Mollar de Elche 17	Pink-yellow	Light pink	C/C	-
P.G.202-213	XBS	Pink-yellow	White	C/C	-
P.G.167-68	"orange"	Orange-yellow	White	C/C	-
P.G.219-230	SP1	Red	Dark red	C/C	-
P.G.222-233	SP4	Red	Red	C/C	-

Further genomic DNA sequencing of *PgLDOX* indicated that the "white" pomegranate contains an insertion between positions 90–91 downstream of the ATG initiation codon (Fig 6). This was deduced from the inability to generate amplified fragment from the genomic DNA of the "white" pomegranate using a set of primers positioned along the *PgLDOX* gene. The precise location of the insertion was initially deduced by comparison of the PCR fragments amplified from red accessions to those amplified from "white" accessions. It was found that only primer sets which flanked the site positioned at 90–91 did not generate a PCR fragment, from the genomic DNA of the "white" pomegranate. Using PCR with a primer set flanking the insertion in the "white" pomegranate; it was possible to distinguish between different pomegranate accessions from the collection. While the colored accessions from the collection and the colored siblings of the "white" pomegranate gave a 240-bp band, no band was obtained from the "white" pomegranate. This was due to the insertion that made the product too long for amplification with our standard procedures. Therefore, the "white" pomegranate contains this insertion in both alleles of *PgLDOX* whereas the examined colored accessions did not have this insertion in any of the alleles of *PgLDOX* (Table 2 and S4 Fig).

All our efforts to amplify the insertion and measuring the size of the insertion in the "white" pomegranate did not succeed. Four kinds of kits that contain a thermostable Taq DNA polymerase and allow the amplification of long or difficult (high GC content) templates were used: Expand Long Template PCR System (Roche), Color Perpetual OptiTaq DNA Polymerase (EURx), Q5 High-Fidelity DNA Polymerase (*BioLabs*) and TaKaRa LA Taq^®^ DNA Polymerase (Clontech). None of the kits enabled the amplification the insert. The inability to amplify the insert could be due to the very large size of the insert or an unusual sequence of nucleotides in the insert that might contain repeats and duplications. However, a short sequence (18 bp) from the insert was obtained by using an AFLP approach with a specific primer from the *PgLDOX* gene (S5 Fig). Using a primer based on the partial sequence obtained by the AFLP analysis in combination with a primer from the *PgLDOX* gene yielded a fragment of DNA which confirmed the presence of the insertion in the "white" pomegranate and in the F2 progeny which showed the "white" phenotype. All of the plants with a "white" phenotype contained this insertion in both alleles of *PgLDOX*, whereas the red F2 progeny did have at least one allele of *PgLDOX* without the insertion (please see more details in the following sub-section).

### Establishment of a segregating F2 population for the "white" phenotype

To assess whether the insertion in *PgLDOX* is segregating for the "white" phenotype, populations from a cross between a colored accession ('Nana1') and the "white" pomegranate were evaluated. The F1 population contained 79 progeny, none of which showed the "white" phenotype. All of the F1 progeny had colored flowers, pink/red skin and arils and red young leaves. Self-pollination of randomly selected progeny from the F1 population generated five F2 populations with a total of 464 progeny. The F2 population segregated for the "white" phenotype with 27% of the progeny being "white" (126 out of 464). These results clearly indicate that the "white" mutation is recessive and has a single-gene Mendelian type of inheritance (confirmed by Chi-square test, *P (Χ* ^*2*^ *≤* *0*.*72)*).

**Fig 7:**
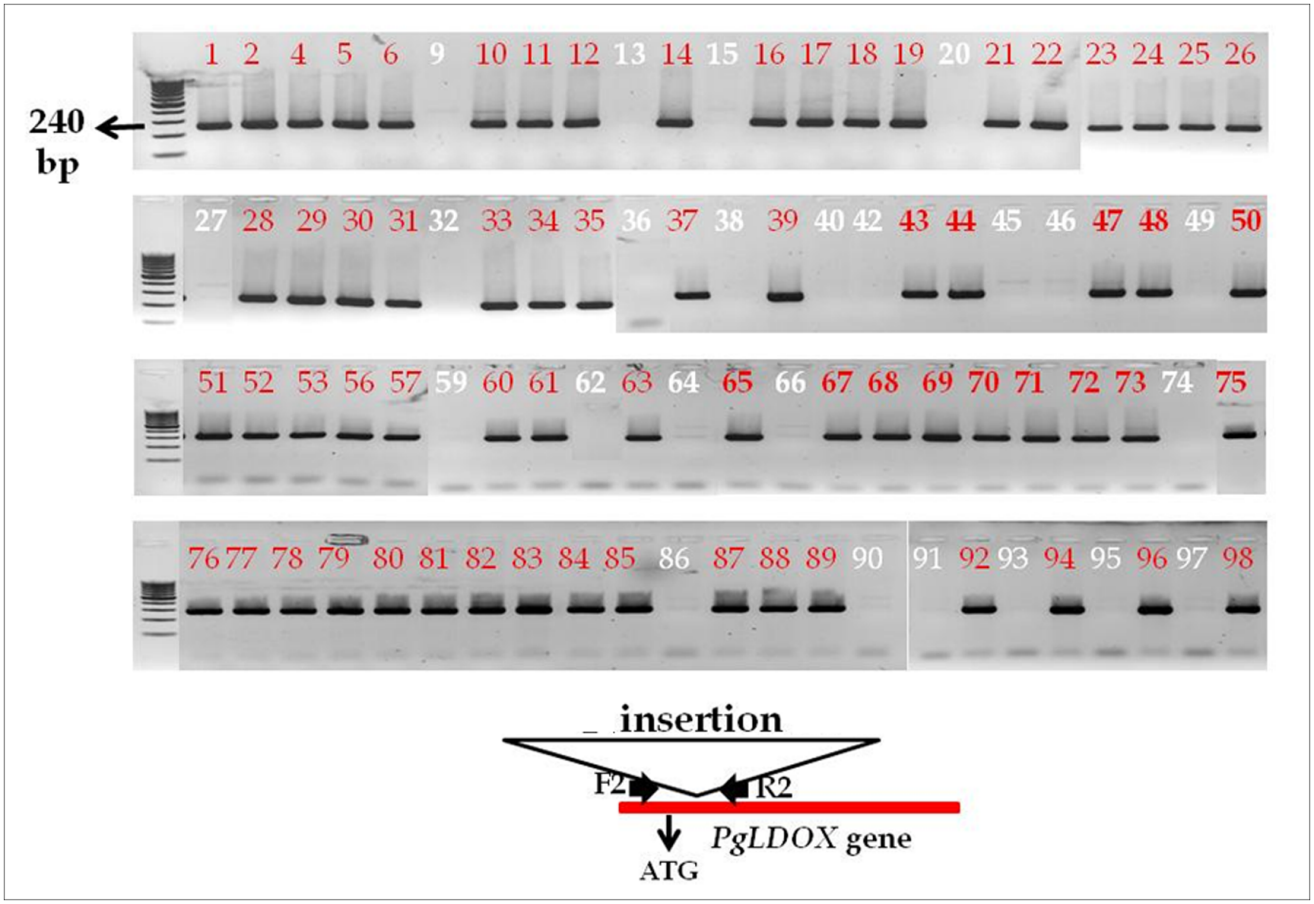
PCR amplification for the absence of the insertion within the *PgLDOX* gene in the F2 progeny. White numbers indicate progeny with "white" phenotype and red numbers indicate progeny with colored phenotype. A scheme of the location of the primers (F2 and R2) with relevance to the insert is in the bottom. PCR products were separated in a 1% agarose gel.

Using PCR with a primer set flanking the insertion in the "white" phenotype; it was possible to distinguish between the two parents of the population. While the colored accession 'Nana1' gave a 240-bp band, no band was obtained from the "white" pomegranate. This analysis was used to genotype 91 progeny of one of the F2 populations. All of the "white" progeny did not gave a 240-bp band, means that they contained the insertion in both alleles of *PgLDOX*, whereas all of the colored progeny gave a 240-bp band, means that they have at least one alleles of *PgLDOX* without the insertion (Fig 7 and S3 Table).

**Fig 8:**
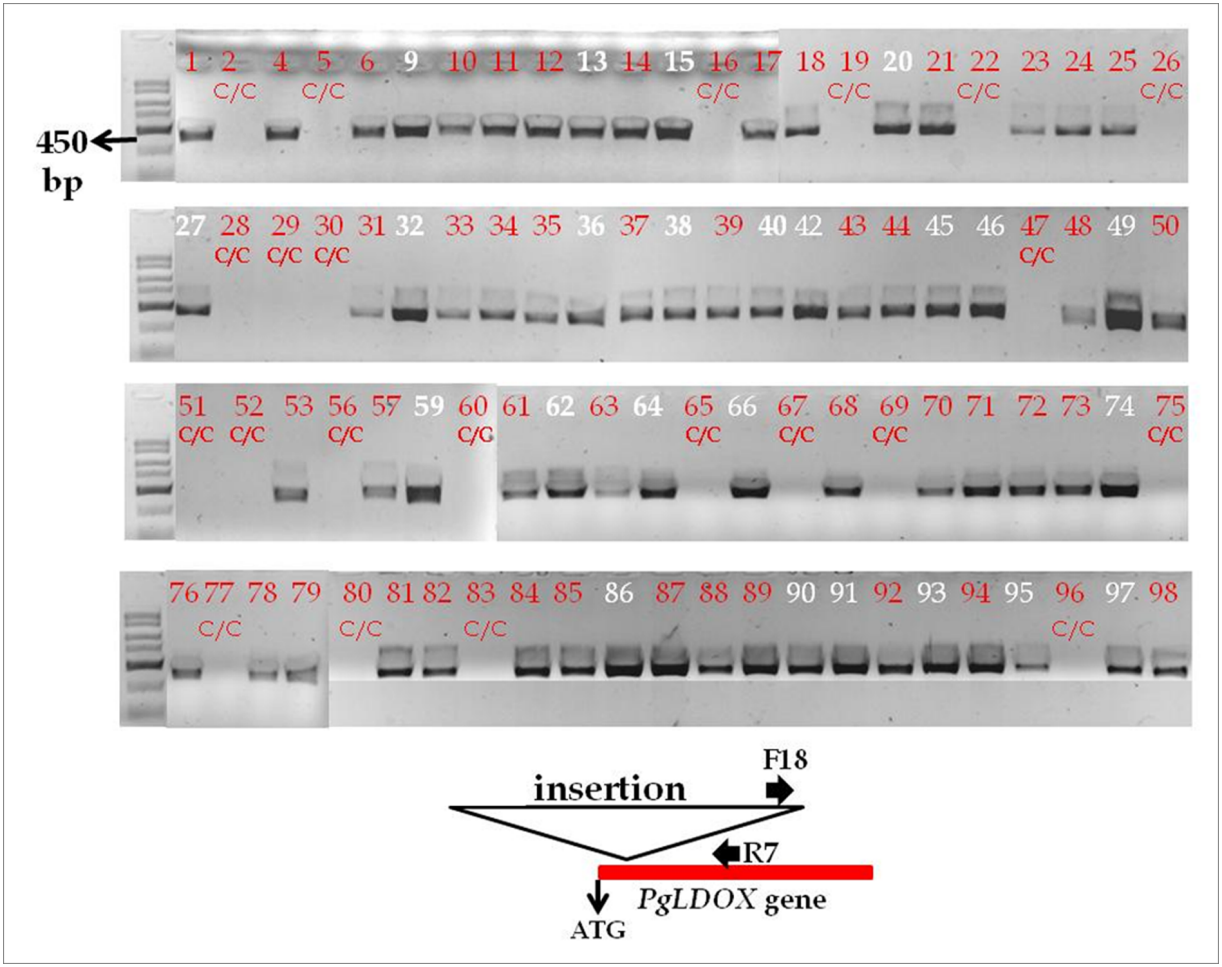
Co-dominance primer markers that distinguish between heterozygote and homozygote alleles for the insert in the red F2 progeny. White numbers indicate progeny with "white" phenotype and red numbers indicate progeny with colored phenotype. The red lettering C/C indicates homozygocity for the SNP marker (1,008 bp downstream to the ATG, shown also in S3 Table). A scheme of the location of the F18 and R7 primers with relevance to the insert is in the bottom (F18 primer is based on the 18bp that were sequencing from the insert, using the AFLP method). PCR products were separated in a 1% agarose gel.

Using the primer based on the partial sequence from the insertion it was possible to obtain positive data for the presence of the insertion. Indeed, when the population was screened with primer combination containing the primer based on the insert and a primer from the *PgLDOX* it was possible to show that all "white" progeny gave a 450-bp band, means that they contained the insertion in both alleles of *PgLDOX*. In addition, using the same primer combination it was also possible to distinguish between heterozygotes for the insertion in the colored F2 progeny which also gave a 450-bp band (Fig 8).

We also used the SNP found in position 1,008 relative to the ATG of *PgLDOX* to screen the population. Sequence data indicated that the colored 'Nana1' accession is homozygous for the "C" allele and the "white" pomegranate is homozygous for the "T" allele. As already noted, it was assumed that this SNP is not responsible for the "white" mutation; however, it could be quite useful as a genetic marker for the parental source and to analyze the segregation of *PgLDOX* in selected populations, such as those described above. All of the F1 progeny showed heterozygosity for the SNP, containing C/T alleles; 24 progeny (26%) of the F2 population contained the T/T alleles, 45 progeny (50%) contained the C/T alleles, and 22 progeny (24%) contained the C/C alleles. All of the "white" progeny segregated with the T/T genotype (S3 Table). The data clearly show that the "white" phenotype segregates with the *PgLDOX* alleles of the "white" pomegranate.

## Discussion

Color plays an important role in pomegranate's commercial value as consumers and the industry demand fruits with intensely red-colored skin and arils. Moreover, pigments such as anthocyanins are known for their health-promoting effects. In this study, the molecular-genetic control of color production was investigated in the "white" pomegranate accession. Elucidating the molecular cause for the "white" color could shed light on color control in pomegranate, and enhance our ability to evaluate the impact of different compositions of anthocyanin derivatives and other flavonoids on the human diet, protection from environmental cues such as radiation and heat, and shelf life. In addition, the white flower mutations might be used for breeding ornamental cultivars and as visible genetic markers in breeding.

### Characterization of white flowers and fruits in plant species

White-colored phenotypes are known in various non-fruit-tree species such as strawberry [44–46], mock strawberry [47], gentian flowers [48], dahlia [49–50], Arctic mustard flowers [51], petunia [52] and *Matthiola incana* [53], among others. In mock strawberry, the white phenotype is suggested to be due to low expression of *ANS/LDOX*, but the gene responsible for the phenotype was never conclusively determined. In the other abovementioned plant species, the white phenotype was suggested to be due to a MYB factor (strawberry and gentian flowers), bHLH (dahlia), TTG1-like (petunia and *M*. *incana*) or CHS (dahlia and Arctic mustard flowers). These examples show that the causes for the white phenotype can be manifold in different plant species.

There are a few studies describing white mutants in fruit trees, including grape and Malay apple. It has been shown that the difference between red and white grapes is due to mutations in two MYB transcription factors which specifically control UFGT expression [54–55]. In the white Malay apple, it was suggested that lack of *MYB* expression is part of the reason for the lack of UFGT expression and anthocyanin synthesis during fruit development [56]. Whereas in both grape and Malay apple, a change in a regulatory gene (MYB-type) was shown to cause the white phenotype, in pomegranate, the "white" phenotype is caused by an insertion in a single structural gene, *PgLDOX*. Recently, Zhao et al. [57] described a white pomegranate and suggested that the white phenotype is due to lack of *LDOX* expression. These results are in agreement with the data presented in this study although Zhao et al. conclusions were based only on expression data and their work was done only on the fruit skin tissue. Our study utilized genetic and chemical data to pinpoint the *PgLDOX* gene as the site and nature of the mutation.

### The flavonoid-biosynthesis pathway in the "white" pomegranate is blocked in the LDOX stage

Using molecular, chemical and genetic analyses, this study shows that the "white" pomegranate is mutated in the gene *PgLDOX* which is involved in anthocyanin biosynthesis in pomegranate flowers, fruits and leaves.

Gene expression analysis indicated that out of 11 genes predicted to be involved in anthocyanin biosynthesis, the structural gene *PgLDOX* is not expressed in any of the "white" pomegranate tissues (Figs 3 and 4). On the other hand, the other examined structural and regulatory genes were expressed. *LDOX* encodes the enzyme LDOX which is also called anthocyanidin synthase (ANS). LDOX catalyzes the conversion of colorless leucoanthocyanidins to the colored anthocyanidins by an oxidation reaction, which is one of the central steps in the anthocyanin and proanthocyanidin pathway [58–60].

In parallel to the molecular analysis, HPLC analysis was performed to determine the composition and amount of anthocyanins and other metabolites in the flavonoid pathway in young leaves, fruits skin, arils and flowers. Our results revealed that in the "white" pomegranate, the ability to produce anthocyanin is completely abolished in all of the examined tissues. The skin of mature fruits of the "white" pomegranate was also found to accumulate significantly higher levels of kaempferol, quercetin, and catechins and their derivatives than the red pomegranate accessions (Fig 5). The findings that the "white" pomegranate can produce flavonoids which correspond to biochemical functions upstream of, but not downstream of *PgLDOX*, and is unable to produce mRNA *PgLDOX*, strongly support the suggestion that the flavonoid pathway in "white" pomegranate is blocked at the *LDOX* stage. As a result, the "white" pomegranate does not produce or accumulate anthocyanins and lacks color as compared to other typical pomegranate accessions.

### The insertion in the *PgLDOX* gene is responsible for the "white" phenotype

Comparison of the *PgLDOX* sequences of "white" pomegranate and different colored pomegranates from the collection revealed two changes in this gene's coding region: a SNP ("T" in the "white" pomegranate and "C" in the colored pomegranates) and an insertion in the "white" pomegranate. By studying populations from a cross between a colored accession and the "white" pomegranate, the inheritance of the "white" phenotype and the linkage between the SNP and insertion markers and the "white" phenotype could be determined. Complete linkage between the segregation of the "white" phenotype and these two markers in *PgLDOX* gene was found. All progeny with "white" phenotype (100%) contained the T/T alleles and contained the insertion in both alleles. All of the colored progeny (100%) contained the C/C or C/T alleles: the heterozygote progeny (C/T) contained the insert only in the allele originating from the "white" pomegranate (T allele) and no insertion was detected in the homozygote (C/C) progeny (Figs 7 and 8). The genetic study of the segregating F2 population strongly supports the assumption that the mutation responsible for the "white" phenotype is in the *PgLDOX* gene.

To determine whether the SNP, the insertion, or both are responsible for the "white" phenotype, additional colored accessions were genotyped from our pomegranate collection. Some red siblings were found to be homozygous for the "white" allele (T/T) in the *PgLDOX* gene, but did not contain the insertion (S4 Fig). Based on these findings, it is concluded that the cause of the "white" anthocyanin-less pomegranate phenotype is a mutation consisting of the insertion in the ORF of the *LDOX* gene. Since the insertion is located in the coding region at position 90 from the ATG, it disrupts the transcription of the normal gene and is likely to generate an instable chimeric abnormal transcript that contains only the first 90 bp from the ATG of *PgLDOX*. This assumption is verified by the absence of any *PgLDOX* transcript in the "white" pomegranate that contains the *PgLDOX* gene sequence. Hence, no active LDOX enzyme is produced in the "white" pomegranate. Furthermore, in the "white" pomegranate, a lack of anthocyanins was shown in all tested parts of the plant, including the flowers, fruits and young leaves. This phenotype strengthens the assumptions that a single *LDOX* gene is responsible for the production of anthocyanins in pomegranate and that *PgLDOX* encodes the major LDOX in pomegranate.

### The function of LDOX in plants

The role of *LDOX* in the anthocyanin-biosynthesis pathway has been characterized in many plants, including: Arabidopsis [61,62], apple [63], *Torenia hybrida* flower [64], onion [65–66], grape [67], Chinese bayberry [68], and strawberry [69]. In apple silencing of LDOX in a red-leafed apple cultivar caused almost complete blockage of anthocyanin biosynthesis. This was accompanied by a shift in the profile of the flavonoids and related polyphenols [63]. Like in apple, inactivation of LDOX in pomegranate resulted in blockage of anthocyanin biosynthesis and a shift in the accumulation of intermediate flavonoids.

The potential roles of LDOX in peach fruit storage [70] and in transgenic apple tree survival [63] are examples to the potential of LDOX in control of important agricultural traits. However, since the "white" phenotype, which lacks anthocyanins in all tested tissues, is viable and grows in open orchards, it is concluded that pomegranate plants can survive without anthocyanins. It should be emphasized, however, that although the anthocyanin-less fruit develops normally, it is highly susceptible to sunburn and browning.

Identification of the genetic components responsible for the observed color variability in fruit trees is particularly complicated, mostly because there are no adequate genetic systems to analyze the genetic variability in fruit trees. Pomegranate, among others, could be developed into such a system, because it has a relatively short juvenile period and it is easy to efficiently establish crossbred populations [7]. In this study, these advantages were combined with a genetic approach to reveal the mechanism responsible for the "white" anthocyanin-less phenotype.

## Supporting Information

S1 Fig Phenotypic characterization of the "white" pomegranate and the red cv. Wonderful. Different developmental stages of the fruit, from flower (stage 1) to fully mature fruit (stage 12) and young leaves from **(A)** the "white" pomegranate accession P.G.254-265 and **(B)** the red cv. Wonderful P.G.100-1.
(TIF)

S2 Fig Multiple alignments of deduced amino acid sequences corresponding to plant gene homologues of pomegranate PgPAL-4 (a); PgCHS-3 (b); PgCHI (c); PgF3H-2 (d); PgF3'H (e); PgF3'5'H (f) and PgLDOX (g) proteins. High consensus residues are highlighted in red color, low consensus residues are highlighted in blue color and neutral residues are highlighted in black color. A multiple sequence alignment was constructed using MultAlin software [38].
(PDF)

S3 Fig Phylogenetic tree showing clustering of PgLDOX and its homologs from other plants. (PDF)

S4 Fig Inability of the SNP in *PgLDOX* to discriminate between the "white" and the colored phenotype. Comparison between SNP data and insertion data in two groups of pomegranate: siblings from the sub-family (in left) and different colored accessions from the pomegranate collection (in right). The results of SNP analysis are presented in T/T or C/C lettering. The PCR analysis for the detection of the insertion is presented as bands separated on 1% agarose gel.
(TIF)

S5 Fig Scheme and data from the AFLP analysis. The various steps (as detailed in materials and methods) involved in the AFLP analysis are displayed.
(TIF)

S1 Table Primers used for semi-quantitative RT-PCR experiments. (DOCX)

S2 Table List of primers used in this work and their specific role. (DOCX)

S3 Table Phenotype and genotype segregation analysis of the F2 population. (DOCX)

## Abbreviations

HPLC: High performance liquid chromatography
LDOX: Leucoanthocyanidin dioxygenase
PG: *Punica granatum*
SNP: Single Nucleotide Polymorphism
