# Supplementary figures and images for: A "White" Anthocyanin-less Pomegranate (Punica granatum L.) Caused by an Insertion in the Coding Region of the Leucoanthocyanidin Dioxygenase (LDOX; ANS) Gene
Source: PLoS One. 2015 Nov 18;10(11):e0142777. doi: 10.1371/journal.pone.0142777 (PMC4651307; doi:10.1371/journal.pone.0142777)

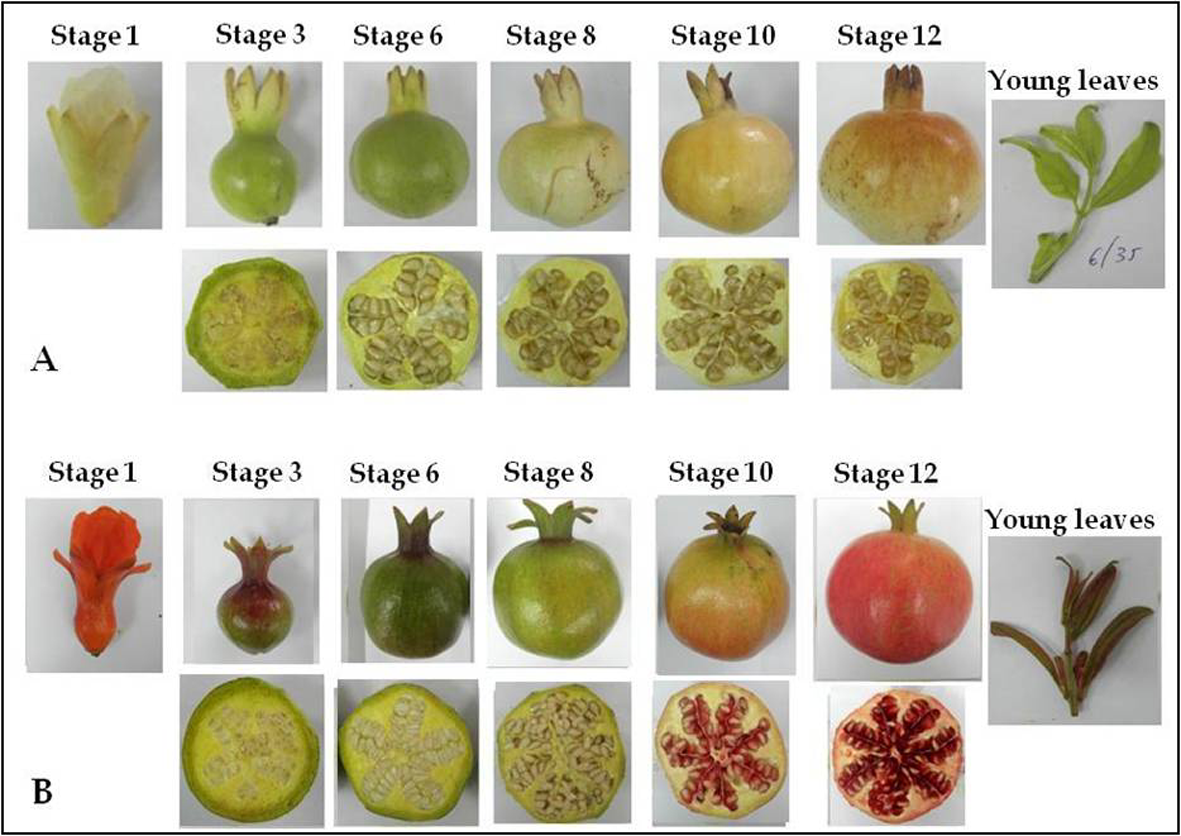

Supplement: S1 Fig — Different developmental stages of the fruit, from flower (stage 1) to fully mature fruit (stage 12) and young leaves from (A) the "white" pomegranate accession P.G.254-265 and (B) the red cv. Wonderful P.G.100-1. (TIF) [file pone.0142777.s001.tif]

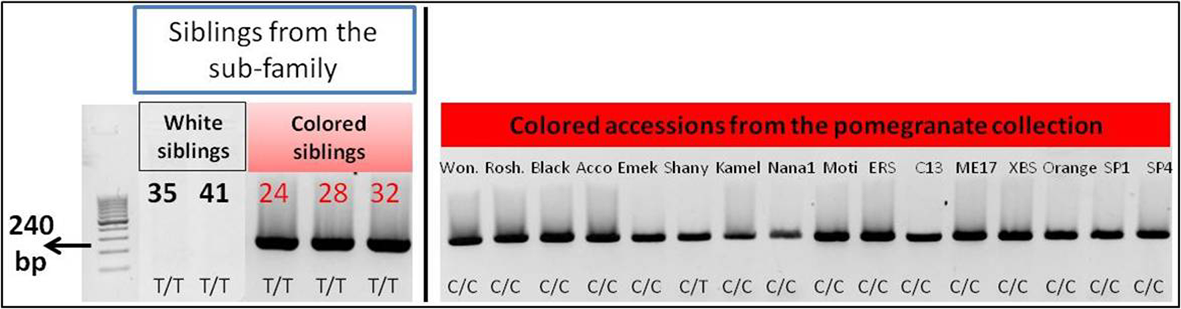

Supplement: S4 Fig — Comparison between SNP data and insertion data in two groups of pomegranate: siblings from the sub-family (in left) and different colored accessions from the pomegranate collection (in right). The results of SNP analysis are presented in T/T or C/C lettering. The PCR analysis for the detection of the insertion is presented as bands separated on 1% agarose gel. (TIF) [file pone.0142777.s004.tif]

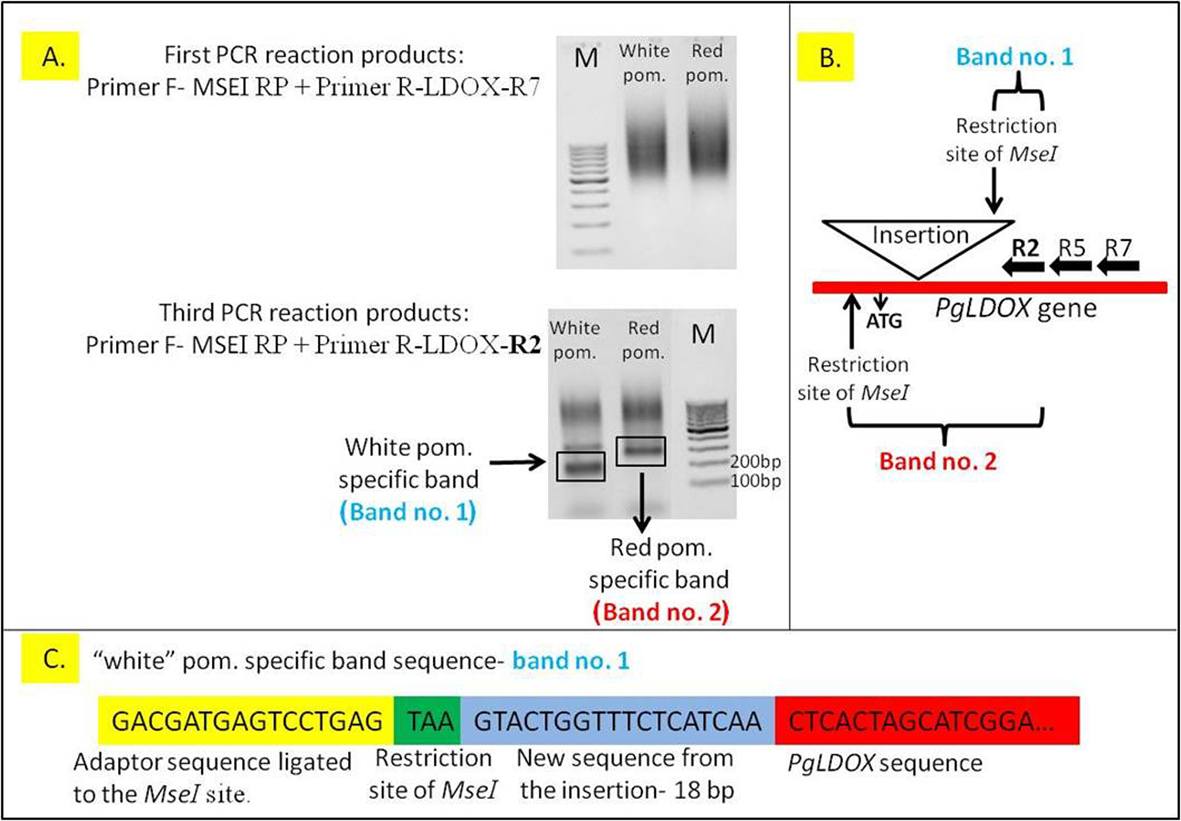

Supplement: S5 Fig — The various steps (as detailed in materials and methods) involved in the AFLP analysis are displayed. (TIF) [file pone.0142777.s005.tif]
