# Supplementary material for: A "White" Anthocyanin-less Pomegranate (Punica granatum L.) Caused by an Insertion in the Coding Region of the Leucoanthocyanidin Dioxygenase (LDOX; ANS) Gene
Source: PLoS One. 2015 Nov 18;10(11):e0142777. doi: 10.1371/journal.pone.0142777 (PMC4651307; doi:10.1371/journal.pone.0142777)
Supplement: S2 Fig — High consensus residues are highlighted in red color, low consensus residues are highlighted in blue color and neutral residues are highlighted in black color. A multiple sequence alignment was constructed using MultAlin software [38]. (PDF) [file pone.0142777.s002.pdf]

S2 Fig. Multiple alignments of deduced amino acid sequences corresponding to plant gene homologues of pomegranate PgPAL-4 (a); PgCHS-3 (b); PgCHI (c); PgF3H-2 (d); PgF3'H (e); PgF3'5'H (f) and PgLDOX (g) proteins.

(a) PAL

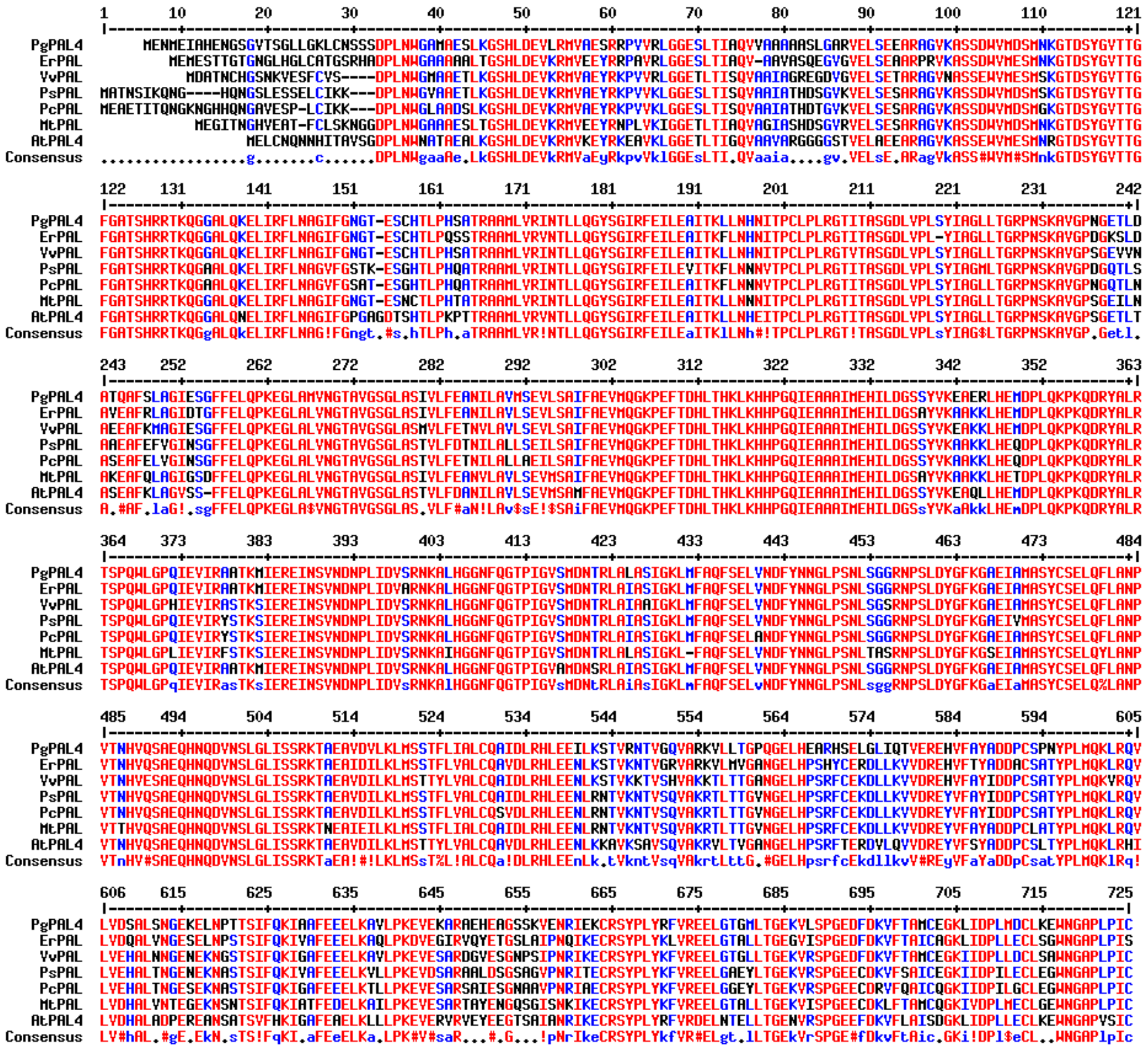

PgPAL-4 from *Punica granatum* (pomegranate) (this work, Ophir *et al.*, 2014); ErPAL from *Eucalyptus robusta* (BAL49995.1); VvPAL from *Vitis vinifera* (grapevine) (ABM67591.1); PsPAL from *Prunus salicina* (plum) (AFP24940.1); PcPAL from *Pyrus communis* (pear) (AGL50914.1); MtPAL from *Medicago truncatula* (XP_003590471.1); AtPAL4 from *Arabidopsis thaliana* (NP_187645.1).

(b) CHS

```
           1        10        20        30        40        50        60        70        80        90       100
           |--------+---------+---------+---------+---------+---------+---------+---------+---------+---------|
  PgCHS-3       MVTVEEVCRAQRAEGPATIMAIGTATPPNCVDQSTYPDYYFRITNSEHKTELKEKFKRMCEKSMIKKRYMYLTEELLKENPNVCAYMAPSLDARQ
    GhCHS       MVTVEEVRKAQRAQGPATVLAIGTSTPPNCVDQSTYPDYYFRITNSEHKTELKEKFKRMCEKSMIKKRYMYLTEEILKENPNVCEYMAPSLDARQ
    MdCHS       MVTVEEVRKAQRAEGPATVMAIGTATPSNCVDQATYPDYYFRITNSEHKVELKEKFQRMCDKSMIKKRYMYLTEEILKENPSVCEYMAPSIDARQ
    PhCHS       MVTVEEIRRAQRAEGPATIMAIGTATPSNCVDQSTYPDYYFRITNSEHKTELKEKFQRMCDKSMIKKRYMHLTEEILKENPNICEYMAPSLDARQ
    VvCHS       MVSVAEIRKAQRAEGPATVLAIGTATPANCVYQADYPDYYFRITNSEHMTELKEKFKRMCEKSMINKRYMHLTEEILKENPNVCAYMAPSLDARQ
    AtCHS  MVMAGASSLDEIRQAQRADGPAGILAIGTANPENHVLQAEYPDYYFRITNSEHMTDLKEKFKRMCDKSTIRKRHMHLTEEFLKENPHMCAYMAPSLDTRQ
Consensus  .....mvtveE!r.AQRA#GPAt!$AIGTatP.NcVdQatYPDYYFRITNSEHkt#LKEKFkRMC#KSmIkKRyMhLTEEiLKENPnvCaYMAPSlDaRQ

           101      110       120       130       140       150       160       170       180       190      200
           |--------+---------+---------+---------+---------+---------+---------+---------+---------+---------|
  PgCHS-3  DIVVVEVPKLGKEAAQKAIKEWGQPKSKITHLVFCTTSGVDMPGADYQLTKLLGLRPSVKRFMMYQQGCFAGGTVLRMAKDLAENNKGARVLVVCSEITA
    GhCHS  DMVVVEVPKLGKEAATKAIKEWGQPKSKITHLVFCTTSGVDMPGADYQLTKLLGLRPSVKRLMMYQQGCFAGGTVLRVAKDLAENNKGARVLVVCSEITA
    MdCHS  DMVVVEVPKLGKEAATKAIKEWGQPKSKITHLVFCTTSGVDMPGADYQLTKLLGLRPSVKRLMMYQQGCFAGGTVLRLAKDLAENNKGARVLVVCSEITA
    PhCHS  DIVVVEVPKLGKEAAQKAIKEWGQPKSKITHLVFCTTSGVDMPGADYQLTKLLGLRSSVKRLMMYQQGCFAGGTVLRLAKDLAENNKGARVLVVCSEITA
    VvCHS  DMVVVEVPKLGKEAAVKAIKEWGQPKSKITHLVFCTTSGVDMPGADYQLTKLLGLKPSVKRLMMYQQGCFAGGTVLRLAKDLAENNAGSRVLVVCSEITA
    AtCHS  DIVVVEVPKLGKEAAVKAIKEWGQPKSKITHVVFCTTSGVDMPGADYQLTKLLGLRPSVKRLMMYQQGCFAGGTVLRIAKDLAENNRGARVLVVCSEITA
Consensus  DiVVVEVPKLGKEAA.KAIKEWGQPKSKITHlVFCTTSGVDMPGADYQLTKLLGLrpSVKRlMMYQQGCFAGGTVLR.AKDLAENNkGaRVLVVCSEITA

           201      210       220       230       240       250       260       270       280       290      300
           |--------+---------+---------+---------+---------+---------+---------+---------+---------+---------|
  PgCHS-3  VTFRGPSDTHLDSLVGQALFGDGSAAVIVGSDPIPGV-EKPLYQLVSVAQTILPDSDGAIDGHLRELGLTFHLLKDVPGLISKNIEKSLVEAFQPLGISD
    GhCHS  VTFRGPSDTHLDSLVGQALFGDGAAAVIIGADPVPEI-EKPMFELVSAAQTILPDSDGAIDGHLREVGLTFHLLKDVPGLISKNIEKSLVEAFQPLGISD
    MdCHS  VTFRGPSDTHLDSLVGQALFGDGAAAVIIGADPVPEV-EKPLFELVSAAQTILPDSDGAIDGHLREVGLTFHLLKDVPGLISKNIEKSLNEAFKPIGISD
    PhCHS  VTFRGPNDTHLDSLVGQALFGDGAAAIIIGSDPLPGV-ERPLFELVSASQTLLPDSEGAIDGHLREVGLTFHLLKDVPGLISKNIQKSLVEAFQPLGISD
    VvCHS  VTFRGPSDTHLDSLVGQALFGDGAAAVIIGADPDTKI-ERPLFELVSAAQTILPDSEGAIDGHLREVGLTFHLLKDVPGLISKNIEKSLVEAFTPIGISD
    AtCHS  VTFRGPSDTHLDSLVGQALFSDGAAALIVGSDPDTSVGEKPIFEMVSAAQTILPDSDGAIDGHLREVGLTFHLLKDVPGLISKNIVKSLDEAFKPLGISD
Consensus  VTFRGPsDTHLDSLVGQALFgDGaAAvI!GsDP.p.!.EkPl%#$VSaaQTiLPDS#GAIDGHLREvGLTFHLLKDVPGLISKNIeKSLvEAF.PlGISD

           301      310       320       330       340       350       360       370       380       390     399
           |--------+---------+---------+---------+---------+---------+---------+---------+---------+--------|
  PgCHS-3  WNSIFWIAHPGGKAILDQVEVKLGLKPEKLRATRHVLSEYGNMSSACVLFILDEMRKKCVEDGLRTTGEGLDWGVLFGFGPGLTVETVVLHSVGLAH
    GhCHS  WNSLFWIAHPGGPAILDQVEAKLALKPEKLRATRHVLSEYGNMSSACVLFILDEMRKKSREDGLQTTGEGLEWGVLFGFGPGLTVETVVLHSVAA
    MdCHS  WNSLFWIAHPGGPAILDQVEAKLALKPEKLEATRQVLSDYGNMSSACVLFILDEVRRKSAEKGLKTTGEGLEWGVLFGFGPGLTVETVVLHSVGLTA
    PhCHS  WNSIFWIAHPGGPAILDQVELKLGLKPEKLRATRHVLSEYGNMSSACVLFILDEMRKASSKEGLGTTGEGLEWGVLFGFGPGLTVETVVLHSVST
    VvCHS  WNSLFWIAHPGGPAILDQVELKLGLKEEKLRATRHVLSEYGNMSSACVLFILDEMRKKSIEEGKASTGEGLEWGVLFGFGPGLTVETVVLHSVSAPPAH
    AtCHS  WNSLFWIAHPGGPAILDQVEIKLGLKEEKMRATRHVLSEYGNMSSACVLFILDEMRRKSAKDGVATTGEGLEWGVLFGFGPGLTVETVVLHSVPL
Consensus  WNSlFWIAHPGGpAILDQVE.KLgLKpEK$rATRhVLS#YGNMSSACVLFILDEmRkks.edGl.tTGEGL#WGVLFGFGPGLTVETVVLHSV.l....
```

PgCHS-3 from *Punica granatum* (pomegranate) (this work, Ophir *et al*., 2014); GhCHS from *Gossypium hirsutum* (cotton) (ABS52573.1); MdCHS from *Malus x domestica* (apple) (AAX16492.1); PhCHS from *Petunia hybrida* (P22928.2); VvCHS from *Vitis vinifera* (grapevine) (BAA31259.1); AtCHS from *Arabidopsis thaliana* (NP_196897.1).

(c) CHI

```
           1        10        20        30        40        50        60        70        80        90
           |--------+---------+---------+---------+---------+---------+---------+---------+---------|
    PgCHI     MDSFSHGPDNDSGNRSSSGELLIPPAAKPPGSDKTLFLGGAGVRGLDVDGKFVKYTAIGVYLEEKALPLLATEWKGKSAEELTDTDE
    GhCHI           MSTSLSVTELQVENFTFPPTVKPPGSTKTLFLGGAGERGLEIQGKFIKFTAIGVYLEDSAVNCLGVKWKGKSAVELTESVE
    VvCHI           MSQVPSVTAVQVENVLFPPSVKPPGSTNDLFLGGAGVRGLEIQGKFVKFTAIGVYLENSAVPTLAVKWKGKTVEELADSVD
    PhCHI           MSPPVSVTKMQVENYAFAPTVNPAGSTNTLFLAGAGHRGLEIEGKFVKFTAIGVYLEESAIPFLAEKWKGKTPQELTDSVE
    AtCHI  MSSSNACASPSPFPAVTKLHVDSVTFVPSVKSPASSNPLFLGGAGVRGLDIQGKFVIFTVIGVYLEGNAVPSLSVKWKGKTTEELTESIP
    MdCHI           MAPPPSLAGLQVGATAFPPSVKPPGSSNTLFLGGAGMRGLEIQGNFVKVTAIGVYLEDSAVPLLAVKWKGKTAEELSESVE
Consensus  ..............s.....vg...fpP.vKpPgS.ktLFLGGAGvRGL#!#GkF!k.TaIGVYLE..AvplLavkWKGKsaeELt#s.e

           91      100       110       120       130       140       150       160       170       180
           |--------+---------+---------+---------+---------+---------+---------+---------+---------|
    PgCHI  FFRDIITGPFEKLTQVSFITQLTGKQYSDKVAENCIAFWKSIGAYGASEARAIDWFLDVFKDQSFPPGSSIFFTHLSNGSYVISFSKHES
    GhCHI  FFRDVVTGDFEKFIRVTMILPLTGQQYSEKVSENCVAIWKSLGIYTDAEAKAIEKFIEVFKDENFPPGSSILFTISGQGSLTIGFSKDSS
    VvCHI  FFRDVVTGPFEKFTKVTTILPLTGRQYSDKVSENCVAFWKSVGIYTDAEAKAIEKFNEVLKDETFPPGNSILFTHSPLGALTMSFSKDGS
    PhCHI  FFRDVVTGPFEKFTRVTMILPLTGKQYSEKVAENCVAHWKGIGTYTDDEGRAIEKFLDVFRSETFPPGASIMFTQSPLGLLTISFAKDDS
    AtCHI  FFREIVTGAFEKFIKVTMKLPLTGQQYSEKVTENCVAIWKQLGLYTDCEAKAVEKFLEIFKEETFPPGSSILFALSPTGSLTVAFSKDDS
    MdCHI  FFRDIVTGPFEKFIQVIMILPLTGQQYSEKVSENCVFFWKSVGIYTDLEGKAIEQFIDVFKDQNFPPGASILFTQSPKGSLTTSFSKDAS
Consensus  FFR#!!TGpFEKftqV.milpLTG.QYS#KV.ENC!afWKs.G.Ytd.EakA!#.Fl#!FK##.FPPGsSIlFt.s..GsltisFSKd.S

           181     190       200       210       220       230       240       250
           |--------+---------+---------+---------+---------+---------+---------|
    PgCHI  IPEVGNAVIENNELAEAVLETIIGNGGVSPAARKSLATRLAEFMKLEKIDF
    GhCHI  VPEGGKVVIENKLLANSVLESVIGKNGVSPAAKESLASRLSPLFNDCGADSEKPQS
    VvCHI  LPEVGNAVIENKLLTEAVLESIIGKHGVSPEAKKSLAARLSELFCKEAGDEKIEAEKVAPVAC
    PhCHI  VTGTANAVIENKQLSEAVLESIIGKHGVSPAAKCSVAERVAELLKKSYAEEASVFGKPETEKSTIPVIGV
    AtCHI  IPETGIAVIENKLLAEAVLESIIGKNGVSPGTRLSVAERLSQLMMKNKDEKEVSDHSVEEKLAKEN
    MdCHI  MPEATNAVIENKLLSETVLESIVGKHGVSPATKQSLAARLSQLLNGCK
Consensus  iPE.gnaVIENklLa#aVLEs!!Gk.GVSPaar.SlA.RLselm...k.d................
```

PgCHI from *Punica granatum* (pomegranate) (this work, Ophir *et al.*, 2014); GhCHI from *Gossypium hirsutum* (cotton) (A2IBF8.1); VvCHI from *Vitis vinifera* (grapevine) (P51117.1); PhCHI from *Petunia hybrida* (P11650.1); AtCHI from *Arabidopsis thaliana* (NP_191072.1); MdCHI from *Malus x domestica* (apple) (ACP30360.1).

(d) F3H

```
           1        10        20        30        40        50        60        70        80        90        100
           |--------+---------+---------+---------+---------+---------+---------+---------+---------+---------|
PgF3H-2    MAPPTATTLTALAEEKTLQSRFVRDEDERPKVAYNQFSSEIPVISLDGIDDKAEGSRRFEICKKIVAACEEWGIFQVIDHGIDAKLIGDMTRLAKEFFAL
GhF3H      MAP---STLTALAEEKTLQASFVRDEDERPKVAYNQFSNDIPVISLAGIDD--VDGKRGEICKKIVEACEDWGVFQVVDHGVDTKLVSEMTRFAREFFAL
PhF3H      MAP---STLTALAEEKTLQTSFIRDEDERPKVAYNQFSNEIPIISLEGIDD--ETGKRAEICDKIVKACEDWGVFQVVDHGVDAELISQMTTFAKEFFAL
VvF3H      MAP---TTLTALAGEKTLQSSFVRDEDERPKVAYNDFSNEIPVISLEGIDE--VGGRRDEICRKIVEACEDWGIFQVVNHGVDSNLISEMTRLAREFFAL
MdF3H      MAPAT-TTLTSIAHEKTLQQKFVRDEDERPKVAYNDFSNEIPIISLAGIDE--VEGRRGEICKKIVAACEDWGIFQIVDHGVDAELISEMTGLAREFFAL
AtF3H      MAP---GTLTELAGESKLNSKFVRDEDERPKVAYNVFSDEIPVISLAGIDD--VDGKRGEICRQIVEACENWGIFQVVDHGVDTNLVADMTRLARDFFAL
Consensus  MAP...tTLTalA.EktL#s.F!RDEDERPKVAYN.FSn#IP!ISLaGID#..v.grRgEIC.kIVeACE#WG!FQ!!#HG!D..L!s#MTrlAr#FFAL

           101      110       120       130       140       150       160       170       180       190       200
           |--------+---------+---------+---------+---------+---------+---------+---------+---------+---------|
PgF3H-2    PAEEKLRFDMTGGKKGGFIVSSHLQGEAVQDWREIVTYFAYPIRSRDYTRWPDKPEGWRAVTEEYSEKLMELACKLLGVLSEAMGLEKEALTKACVDMDQ
GhF3H      PAEEKLRFDMSGGKKGGFIVSSHLQGEAVQDWREIVTYFSYPLKSRDYSRWPDKPEGWIEVTKEYSEKLMGLACKLLEVLSEAMGLEKEALTKACVDMDQ
PhF3H      PPEEKLRFDMSGGKKGGFIVSSHLQGEVVQDWREIVTYFSYPTRARDYSRWPDKPEGWIAVTQKYSEKLMELACKLLDVLSEAMGLEKEALTKACVDMDQ
VvF3H      PPEEKLRFDMSGGKKGGFIVSSHLQGEAVQDWREIVTYFSYPLRTRDYSRWPDKPEGWRSVTQEYSEKLMGLACKLLEVLSEAMDLDKDALTNACVDMDQ
MdF3H      PSEEKLRFDMSGGKKGGFIVSSHLQGEAVQDWREIVTYFSYPIRHRDYSRWPDKPEAWREVTKKYSDELMGLACKLLGVLSEAMGLDTEALTKACVDMDQ
AtF3H      PPEDKLRFDMSGGKKGGFIVSSHLQGEAVQDWREIVTYFSYPVRNRDYSRWPNKPEGWVKVTKEYSERLMSLACKLLEVLSEAMGLEKESLTNACVDMDQ
Consensus  PpE#KLRFDMsGGKKGGFIVSSHLQGEaVQDWREIVTYFsYP.r.RDYsRWP#KPEgWr.VTkeYS#kLM.LACKLLeVLSEAMgL#k#aLTkACVDMDQ

           201      210       220       230       240       250       260       270       280       290       300
           |--------+---------+---------+---------+---------+---------+---------+---------+---------+---------|
PgF3H-2    KVVVNFYPKCPQPDLTLGLKRHTDPGTITLLLQDQVGGLQATKDDGKTWITVQPIEGAFVVNLGDHGHFLSNGRFKNADHQAVVNSDCSRLSIATFQNPA
GhF3H      KVVVNFYPKCPQPDLTLGLKRHTDPGTITLLLQDQVGGLQATRDNGKTWITVQPVEGAFVVNLGDHGHYLSNGRFKNADHQAVVNSNCSRLSIATFQNPA
PhF3H      KVVVNFYPKCPEPDLTLGLKRHTDPGTITLLLQDQVGGLQATKDNGKTWITVQPVEGAFVVNLGDHGHFLSNGRFKNADHQAVVNSNSSRLSIATFQNPA
VvF3H      KVVVNFYPQCPQPDLTLGLKRHTDPGTITLLLQDQVGGLQATRDGGKTWITVQPVEGAFVVNLGDHGHYLSNGRFKNADHQAVVNSNHSRLSIATFQNPA
MdF3H      KVVVNFYPKCPQPDLTLGLKRHTDPGTITLLLQDQVGGLQATRDDGKTWITVQPVEGAFVVNLGDHGHLLSNGRFKNADHQAVVNSNSNRLSIATFQNPA
AtF3H      KIVVNYYPKCPQPDLTLGLKRHTDPGTITLLLQDQVGGLQATRDNGKTWITVQPVEGAFVVNLGDHGHFLSNGRFKNADHQAVVNSNSSRLSIATFQNPA
Consensus  K!VVN%YPkCP#PDLTLGLKRHTDPGTITLLLQDQVGGLQATrD.GKTWITVQP!EGAFVVNLGDHGHfLSNGRFKNADHQAVVNS#ssRLSIATFQNPA

           301      310       320       330       340       350       360       370373
           |--------+---------+---------+---------+---------+---------+---------+--|
PgF3H-2    PDAIVYPLKVPEGEKPVMEEPITFAEMYRRKMSKDLEVARLKKLAKEQQQMKEME-SAERLKLETKTLNDILA
GhF3H      PDATVYPLKIREGEKPILEEPITFAEMYRRKMSKDLELARLKKLAKEQQQLKEKEAENEKPKLEAKPLEEILA
PhF3H      PEAIVYPLKIREGEKSIMDEPITFAEMYRRKMSKDLELARLKKQAKEQQLQAEVAAE--KAKLESKPIEEILA
VvF3H      PEATVYPLKIREGEKAVLEGPITFAEMYRRKMSKDLELARLKKLAKEQQLQ-----DVEKAKLESKPIDQILA
MdF3H      QEAIVYPLSVREGEKPILEAPITYTEMYKKKMSKDLELARLKKLSKEQQLQ-----DLEKAKVDTKPVDDIFA
AtF3H      PDATVYPLKVREGEKAILEEPITFAEMYKRKMGRDLELARLKKLAKEERDH----------KEVAKPVDQIFA
Consensus  p#AtVYPLk!rEGEK.!$#ePIT%aEMYrrKMskDLElARLKKlaKE#q.........ek.Kle.Kp.##IlA
```

PgF3H-2 from *Punica granatum* (pomegranate) (this work, Ophir *et al*., 2014); GhF3H from *Gossypium hirsutum* (cotton) (ABM64799.1); PhF3H from *Petunia hybrida* (AAC49929.1); VvF3H from *Vitis vinifera* (grapevine) (XP_002267640.1); MdF3H from *Malus x domestica* (apple) (AAX89397.1); AtF3H from *Arabidopsis thaliana* (AAM65101.1).

(e) F3'H

```
           1       10        20        30        40        50        60        70        80        90        100
           |--------+---------+---------+---------+---------+---------+---------+---------+---------+---------|
  PgF3'H   GETPTTPLVDSLPFFLAAVATIFFVYYLLQFL-LRPAKPLPPGPRPWPLVGNLPHLGPVPHHRIAAMARTYGPLMHLKLGFVDVVVAASASVAAQFLKTH
  VvF3'H          MNPLALIFCTALFCILLYHFL-----TRRSVRLPPGPKPWPIVGNLPHLGPVPHHSIAALAKTYGPLMHLRMGFVDVVVAASASVAAQFLKTH
  MdF3'H            MFVLIVFTVVFAFFLYRIFAPGG-SRHSLPLPPGPKPWPVVGNLPHLGPVPHHSLAALARQYGPLMHLRLGFVDVVVAASASVASQFLKTH
  PhF3'H          MEILSLILYTVIFSFLLQFILRSFFRKRYPLPLPPGPKPWPIIGNLVHLGPKPHQSTAAMAQTYGPLMYLKMGFVDVVVAASASVAAQFLKTH
  GhF3'H            MASFVLYSILSAVFLYFVFITS-RKRRRLPLPPGPKPWPIIGNLPHMSPVPHQGLAAMAKVYGPLMHLRLGFVDVVVAASASMAAQFLKVH
  AtF3'H          MATLFLTILLATVLFLILRIFSHRRNRSHNNRLPPGPNPWPIIGNLPHMGTKPHRTLSAMVTTYGPILHLRLGFVDVVVAASKSVAEQFLKIH
Consensus  .......m..l.l....a...fll..if......r...pLPPGPkPWPi!GNLpH$gpvPH..laA$a.tYGPl$hLr$GFVDVVVAASaSvAaQFLKtH

           101     110       120       130       140       150       160       170       180       190       200
           |--------+---------+---------+---------+---------+---------+---------+---------+---------+---------|
  PgF3'H   DTNFLSRPPNSGAKHIAYNYQDLVFAPYGPRWRMLRKISSVHLFSSKALDDFRHIRQEEVTTMIRALARVGPDPVKLGQLLNLCATNALGRVMMGRRVFG
  VvF3'H   DANFSNRPPNSGAKHIAYNYQDLVFAPYGPRWRMLRKICSVHLFSGQALDDFRHIRQEEVLALMRALAREGQTPVKLGQLLNVCTTNALGRVMLGRRVFG
  MdF3'H   DANFSSRPPNSGAKHLAYNYQDLVFAPYGPRWRLLRKISSVHLFSGKALDDLKHVRQEEVGVLAHGLASAGSKPVNLAQLLNVCTVNALGRVMVGRRLFG
  PhF3'H   DANFSSRPPNSGAEHMAYNYQDLVFAPYGPRWRMLRKICSVHLFSTKALDDFRHVRQDEVKTLTRALASAGQKPVKLGQLLNVCTTNALARVMLGKRVFA
  GhF3'H   DSNFSSRPPNAGAKYVAYNYQDLVFAPYGPRWRLLRKMSSLHLFSGKALDDFRQIREEEIRVLVRALASA-KTKVNLGQLLNVCTVNALGQVMMGKRVFG
  AtF3'H   DANFASRPPNSGAKHMAYNYQDLVFAPYGHRWRLLRKISSVHLFSAKALEDFKHVRQEEVGTLTRELVRVGTKPVNLGQLVNMCVVNALGREMIGRRLFG
Consensus  DaNFssRPPNsGAkh.AYNYQDLVFAPYGpRWR$LRKisSvHLFS.kAL#Dfrh!R##E!.t$.raLar.g.kpVnLgQLlNvCtvNALgrvM.GrRvFg

           201     210       220       230       240       250       260       270       280       290       300
           |--------+---------+---------+---------+---------+---------+---------+---------+---------+---------|
  PgF3'H   DGSGQSDAKADEFKSMVVEVMVLAGVFNIGDFVPSLEWLDVQGVAARMKKLHKRFDAFLTAILEEHKAAG--GA--ERHADMLSTLISLK-HSADEEGSN
  VvF3'H   DGSGGEDPKADEFKEMVVELMVLAGVFNIGDFVPALEWLDLQGVASKMKKLHARFDAFLGAIVEEHKISG--SAGSERHVDLLSTLISLK-DNADGEGGK
  MdF3'H   NGMGGEDPKADEFKSMVVEMMVLAGVFNIGDFIPSLEWLDLQGVAGKMKKLHKRFDAFLTAIVEEHKRSR--GG---KHVDMLTTLLSLK-EDADGEGAK
  PhF3'H   DGSGDVDPQAAEFKSMVVEMMVVAGVFNIGDFIPQLNWLDIQGVAAKMKKLHARFDAFLTDILEEHKGKI--FG---EMKDLLSTLISLKNDDADNDGGK
  GhF3'H   DGSGGSDPEADEFKSMVVELMQLAGVFNIGDFIPALEWLDLQGVQAKMKKLHNKFDRFLSAILEEHKTKARQSNGQVKHKDFLSTLISLENVDG-AEGGK
  AtF3'H   ---ADADHKADEFRSMVTEMMALAGVFNIGDFVPSLDWLDLQGVAGKMKRLHKRFDAFLSSILKEHEMN----GQDQKHTDMLSTLISLKGTDLDGDGGS
Consensus  dgsg..DpkAdEFksMVvEmMvlAGVFNIGDF!PsL#WLDlQGVa.kMKkLHkrFDaFL.aIleEHk......g...kh.DmLsTLiSLk..dadg#Ggk

           301     310       320       330       340       350       360       370       380       390       400
           |--------+---------+---------+---------+---------+---------+---------+---------+---------+---------|
  PgF3'H   LTDTEIKALLLNMFTAGTDTTSSTVEWAIAEMLRHPDILARVRDELDSVVGKDRLVTELDLPQLTYLQAVIRENFRLHPPTPLSLPRIAAESCEINGYNI
  VvF3'H   LTDVEIKALLLNLFTAGTDTSSSTVEWAIAELIRHPEMMAQAQQELDAVVGRGRLVTDLDLPKLTYLQAIVKETFRLHPSTPLSLPRMAAESCEINGYHI
  MdF3'H   LTDTEIKALLLNMFTAGTDTSSSTVEWAIAELLRHPKILAQLQQELDQVVGRDRLVTESDLPNLTYLQAVIKETFRLHPSTPLSLPRMATESCEINGFHI
  PhF3'H   LTDTEIKALLLNLFVAGTDTSSSTVEWAIAELIRNPKILAQAQQEIDKVVGRDRLVGELDLAQLTYLEAIVKETFRLHPSTPLSLPRIASESCEINGYFI
  GhF3'H   LSDTEIKALLLNMFTAGTDTSSSTVEWAMAELIRHPNIMAQVRKELDSVVGRDRLVSDLDLPNLTYFQAVIKETFRLHPSTPLSLPRMASDSCDINGYHI
  AtF3'H   LTDTEIKALLLNMFTAGTDTSASTVDWAIAELIRHPDIMVKAQEELDIVVGRDRPVNESDIAQLPYLQAVIKENFRLHPPTPLSLPHIASESCEINGYHI
Consensus  LtDtEIKALLLN$FtAGTDTssSTV#WAiAE$iRhP.i$aqaq.ElD.VVGrdRlV.#lDlpqLtYl#A!!kEtFRLHPsTPLSLPriAs#SC#ING%hI

           401     410       420       430       440       450       460       470       480       490       500
           |--------+---------+---------+---------+---------+---------+---------+---------+---------+---------|
  PgF3'H   PKGSTLLVNVWAIARDPDEWSDPLVFRPERFLPGGEKAGVDVRGNDFELIPFGAGRRICAGMSLGIRMVQLLTATLVHAFDWELANGLMPEKLNMDEAYG
  VvF3'H   PKNATLLVNVWAIARDPEVWEEPLEFRPNRFLPGGERPNADVRGNDFEVIPFGAGRRICAGMSLGLRMVHLLTATLVHAFNWELPEGQVAEKLNMDEAYG
  MdF3'H   PKGATLLVNVWAVSRDPDQWSEPLEFRPERFMSGGEKPNVDIRGNDFEVIPFGAGRRICAGMSLGLRMVSLMTATLVHGFDWTLADGLTPEKLNMDEAYG
  PhF3'H   PKGSTLLLNVWAIARDPNAWADPLEFRPERFLPGGEKPKVDVRGNDFEVIPFGAGRRICAGMNLGIRMVQLMIATLIHAFNWDLVSGQLPEMLNMEEAYG
  GhF3'H   PKGATLLVNVWAISRDPNEWNNPLEFRPERFLPGGERPNADVRGNDFEVIPFGAGRRICAGMSLGLRMVQLLTATLAHAFEWELADGLMPEKLDMEEAYG
  AtF3'H   PKGSTLLTNIWAIARDPDQWSDPLAFKPERFLPGGEKSGVDVKGSDFELIPFGAGRRICAGLSLGLRTIQFLTATLVQGFDWELAGGVTPEKLNMEESYG
Consensus  PKgsTLLvN!WA!aRDP#.Ws#PLeFrP#RF$pGGEkp.vD!rGnDFEvIPFGAGRRICAG$sLGlRm!ql$tATLvhaF#WeLa.G..pEkL#M#EaYG

           501     510       520    527
           |--------+---------+------|
  PgF3'H   LTLQRAEPLLVHPKPRLPRHVY
  VvF3'H   LTLQRAAPLMVHPRPRLSPQVFGK
  MdF3'H   LTLQRAAPLMVHPRNRLAPHAYNASSS
  PhF3'H   LTLQRADPLVVHPRPRLEAQAYIG
  GhF3'H   LTLQRAAPLMVHPRPRLSKHAY
  AtF3'H   LTLQRAVPLVVHPKPRLAPNVYGLGSG
Consensus  LTLQRA.PL.VHPrpRL.p.v%.....
```

PgF3'H from *Punica granatum* (pomegranate) (this work, Ophir *et al.*, 2014); VvF3'H from *Vitis vinifera* (grapevine) (BAE47004.1); MdF3'H from *Malus x domestica* (apple) (ACR14867.1); PhF3'H from *Petunia hybrida* (Q9SBQ9.1); GhF3'H from *Gossypium hirsutum* (cotton) (ADP24158.1); AtF3'H from *Arabidopsis thaliana* (NP_196416.1).

(f) F3'5'H

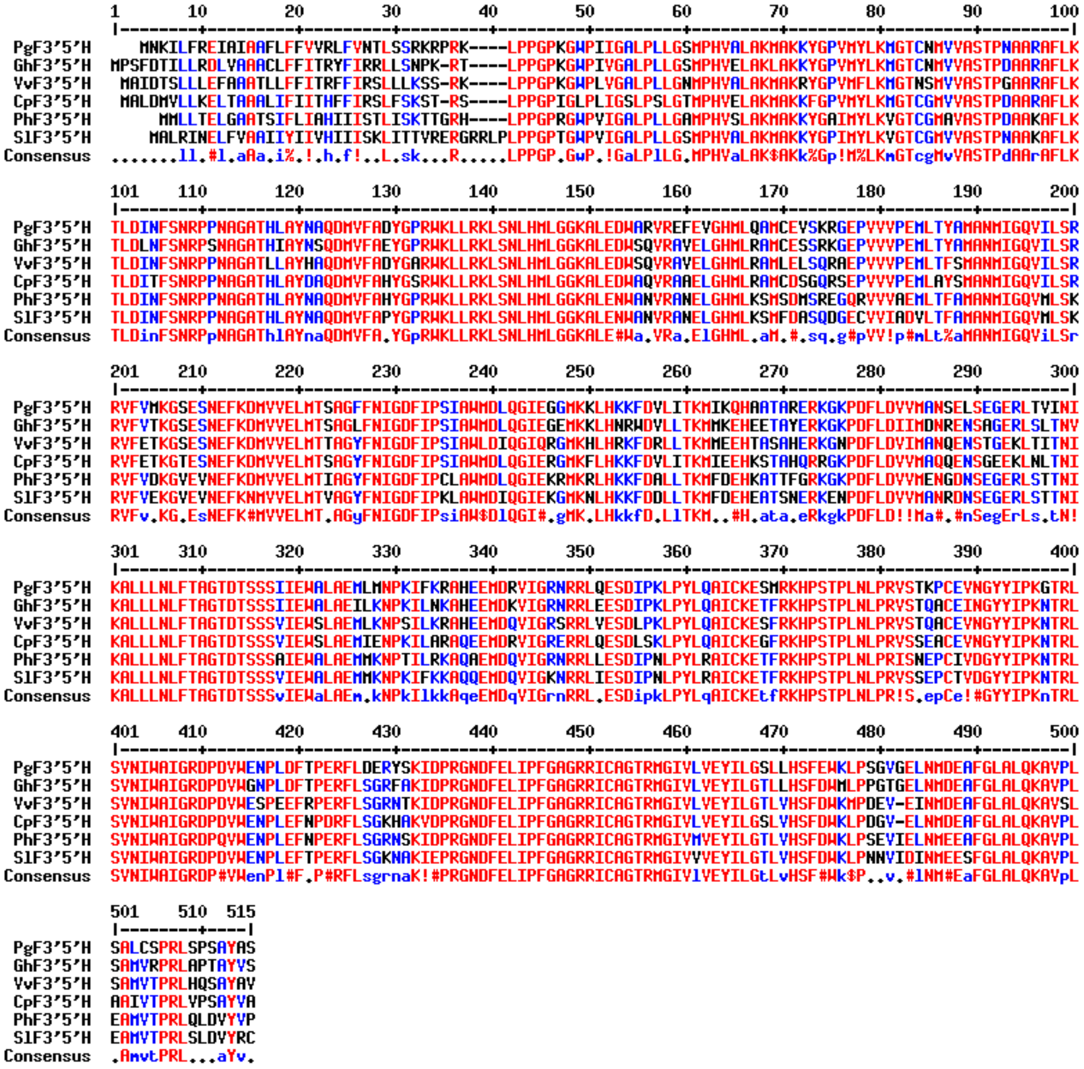

PgF3'5'H from *Punica granatum* (pomegranate) (this work, Ophir *et al*., 2014); GhF3'5'H from *Gossypium hirsutum* (cotton) (AAP31058.1); VvF3'5'H from *Vitis vinifera* (grapevine) (BAE47007.1); CpF3'5'H from *Cyclamen persicum* (ACX37698.1); PhF3'5'H from *Petunia hybrida* (ABN42195.1); SlF3'5'H from Solanum lycopersicum (tomato) (ADC80513.1).

(g) LDOX

```
           1       10        20        30        40        50        60        70        80        90        100
           |--------+---------+---------+---------+---------+---------+---------+---------+---------+---------|
   PgLDOX    MVATVIPRVESLASSGIQSIPKEYVRPEEELTSIGNVFEEEKRDEGPQVPTIDLRDIESEDEVVREKCRAELKKAAADWGVMHLVNHGIPDNLIERVK
   EcLDOX    MVSVVAGRVESLSSSGIQSIPQEYVRPKEELTSIGDIFEEEKKHEGPQVPTIDLEDIASKDPVVRERCHEELRKAATDWGVMHLVNHGIPNDLIERVK
   VvLDOX    MVTSVAPRVESLSSSGIQSIPKEYIRPQEELTSIGNVFEEEKKDEGPQVPTIDLKDIESEDEVVRERCREELKKAAMEWGVMHLVNHGISDDLINRVK
   PpLDOX  MVSSDSVNSRVETLSSSGIATIPKEYIRPKEELINISDIFEQEKSTDGPQVPTIDLKEIDSENENVRERCREELKKAAVDWGVMHLVNHGISDELMDRVR
   MdLDOX  MVSSDSVNSRVETLAGSGISTIPKEYIRPKDELVNIGDIFEQEKNNEGPQVPTIDLKEIESDNEKVRAKCREELKKAAVDWGVMHLVNHGISDELMDKVR
   IbLDOX  MVTTITVPSRVERLAGSGIERIPKEYIRPEEERPSIGDIFAEEKMGGGPQVPTVDLKGINSEDLEVREKCREELRKAAVDWGVMHLVNHGIPEELTGRVK
   AtLDOX      MVAVERVESLAKSGIISIPKEYIRPKEELESINDVFLEEKKEDGPQVPTIDLKNIESDDEKIRENCIEELKKASLDWGVMHLINHGIPADLMERVK
Consensus  ......v..RVEsLasSGI.sIPkEY!RPk#El.sIg#!Fe#EK..eGPQVPT!DLk.IeSe#e.!Re.CreELkKAa.#WGVMHL!NHGIpd#L..rVk

           101     110       120       130       140       150       160       170       180       190       200
           |--------+---------+---------+---------+---------+---------+---------+---------+---------+---------|
   PgLDOX  KAGEEFFNLPIEEKEKYANDQASGRIQGYGSKLANNASGQLEWEDYFFHLVFPEDKRNISIWPKIPSDYKAATAEYARLLRALATRVLSALSLGLGLEEG
   EcLDOX  KAGEVFFNLPIEEKEKHANDQGAGKIQGYGSKLANNASGQLEWEDYFFHLVYPEDKRDLSIWPKHPSDYTAATSEYAKLLRQLATKILSALSISLGLEEG
   VvLDOX  VAGETFFNLPMEEKEKYANDQASGKIAGYGSKLANNASGQLEWEDYFFHLIFPEDKRDMTIWPKTPSDYVPATCEYSVKLRSLATKILSVLSLGLGLEEG
   PpLDOX  KAGKAFFDLPIEQKEKYANDQASGKIQGYGSKLANNASGQLEWEDYFFHLVYPEDKRDLSIWPQTPADYIEATAEYAKELRALATKVLRVLSLGLGLEEG
   MdLDOX  KAGKAFFDLPIELKEKYANDQASGKIQGYGSKLANNASGQLEWEDYFFHCVYPEDKRDLSIWPQTPADYIEATAEYAKQLRELATKVLKVLSLGLGLDEG
   IbLDOX  AAGEGFFGQPIEEKEKYANDQAAGNVQGYGSKLANNASGQLEWEDYFFHCVFPEDKTDLSIWPKTPSDYIAATREYAKQLRALTTTVLAVLSLGLGLEEG
   AtLDOX  KAGEEFFSLSVEEKEKYANDQATGKIQGYGSKLANNASGQLEWEDYFFHLAYPEEKRDLSIWPKTPSDYIEATSEYAKCLRLLATKVFKALSVGLGLEPD
Consensus  kAGe.FF.lpiEeKEKyANDQasGk!qGYGSKLANNASGQLEWEDYFFHlv%PE#Kr#lsIWPktPsDYi.AT.EYak.LR.LaTk!l.vLSlgLGL#eg

           201     210       220       230       240       250       260       270       280       290       300
           |--------+---------+---------+---------+---------+---------+---------+---------+---------+---------|
   PgLDOX  RLEKEVGGLEEMLLQMKINFYPKCPQPELALGVEAHTDISALTFILHNMVPGLQLFYKGKWVTAKCVPNSIIMHIGDTIEILSNGKYKSILHRGLVNKEK
   EcLDOX  RLEKEVGGLEEMLLQMKINYYPKCPQPELALGVEAHTDVSALTFILHNMVPGLQLFYEGKWVTAKCVPNSIIMHIGDTIEILSNRKYKSILHRGLVNKEK
   VvLDOX  RLEKEVGGMEELLLQKKINYYPKCPQPELALGVEAHTDVSALTFILHNMVPGLQLFYEGKWVTAKCVPNSIIMHIGDTIEILSNGKYKSILHRGLVNKEK
   PpLDOX  RLEKEVGGLEELLLQMKINYYPLCPQPELALGVEAHTDVSALTFILHNMVPGLQLFYEGKWVTAKCVPNSIIMHIGDTIEILSNGKYKSILHRGMVNKEK
   MdLDOX  RLEKEVGGLEELLLQMKINYYPKCPQPELALGVEAHTDVSALTFILHNMVPGLQLFYEGKWVTAKCVPNSIVMHIGDTLEILSNGKYKSILHRGMVNKEK
   IbLDOX  RLEKEVGGMEELLLQMKINYYPKCPQPELALGVEAHTDVSALTFILHNMVPGLQLFYEGKWVTAKCVPNSIIMHVGDTVEILSNGKYKSILHRGVVNREK
   AtLDOX  RLEKEVGGLEELLLQMKINYYPKCPQPELALGVEAHTDVSALTFILHNMVPGLQLFYEGKWVTAKCVPDSIVMHIGDTLEILSNGKYKSILHRGLVNKEK
Consensus  RLEKEVGG$EE$LLQmKIN%YPkCPQPELALGVEAHTD!SALTFILHNMVPGLQLFYeGKWVTAKCVP#SI!MH!GDTiEILSNgKYKSILHRGlVNkEK

           301     310       320       330       340       350       36862
           |--------+---------+---------+---------+---------+---------+-|
   PgLDOX  VRISWAVFCEPPREKIILKPLPELVTEAEPAQFPSRTFAQHIEHKLFRKNNPEEKSNK
   EcLDOX  VRISWAVFCEPPKEKIILKPLPELVTEAEPALFPPRTFAQHIEHKLFRKNQAASK
   VvLDOX  VRISWAVFCEPPKEKIILKPLPETVSETEPPLFPPRTFSQHIQHKLFRKTQEALLSK
   PpLDOX  VRISWAVFCEPPKEKIILKPLPETVSETEPPIFPPRTFAEHIQHKLFRKSQEALLNK
   MdLDOX  VRISWAVFCEPPKEKIILKPLPETVSEEEPAMFPPRTFAEHIQHKLFRKSQGALLPK
   IbLDOX  VRVSWAVFCEPPKEKIILQPLPETVSEADPPRFPPRTFAQHIKHKLFRQTDQEAADTPKPDE
   AtLDOX  VRISWAVFCEPPKDKIVLKPLPEMVSVESPAKFPPRTFAQHIEHKLFGKEQEELVSEKND
Consensus  VR!SWAVFCEPPk#KI!LkPLPEtVse.ePa.FPpRTFa#HI.HKLFrk.#.al........
```

PgLDOX from *Punica granatum* (pomegranate) (this work, Ophir *et al*., 2014); EcLDOX from *Eucalyptus camaldulensis* (EcC029638.30, *E. camaldulensis* Genome Database); VvLDOX from *Vitis vinifera* (grapevine) (NP_001268147.1);PpLDOX from *Prunus persica* (peach) (ABX89941.1); MdLDOX from *Malus x domestica* (apple) (AAZ79374.1); IbLDOX from *Ipomoea batatas* (ADE08370.1); AtLDOX from *Arabidopsis thaliana* (NP_194019.1).
