# Supplementary material for: A "White" Anthocyanin-less Pomegranate (Punica granatum L.) Caused by an Insertion in the Coding Region of the Leucoanthocyanidin Dioxygenase (LDOX; ANS) Gene
Source: PLoS One. 2015 Nov 18;10(11):e0142777. doi: 10.1371/journal.pone.0142777 (PMC4651307; doi:10.1371/journal.pone.0142777)
Supplement: S3 Fig — (PDF) [file pone.0142777.s003.pdf]

S3 Fig. Phylogenetic tree showing clustering of PgLDOX and its homologs from other plants.

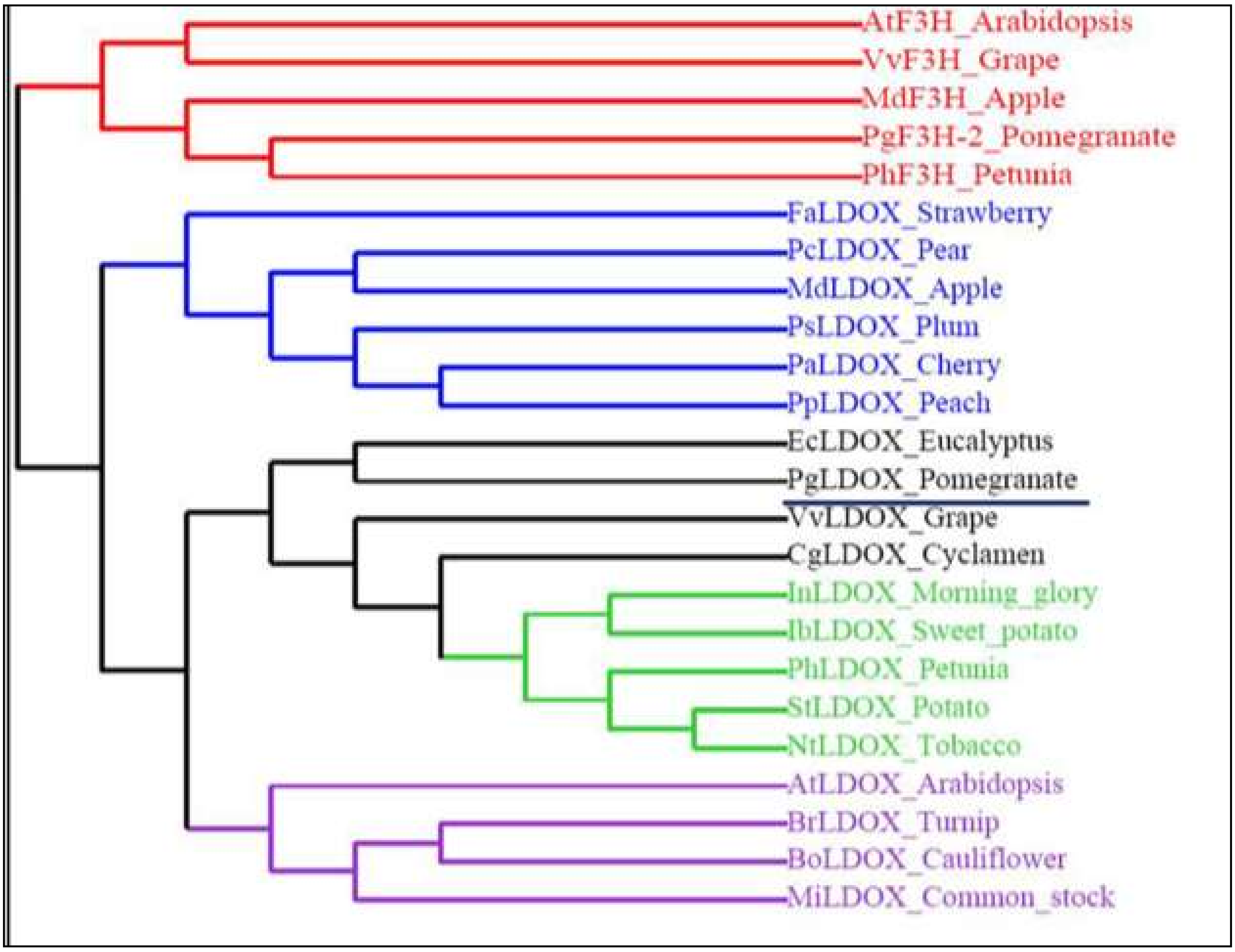

The following LDOX protein accessions are presented, followed by their GenBank accession numbers: *Punica granatum*, pomegranate (PgLDOX; Ophir *et al*., 2014), *Eucalyptus camaldulensis* (EcC029638.30 from *E. camaldulensis* Genome Database), *Cyclamen graecum* (BAJ08044.1), *Vitis vinifera* (NP_001268147.1), *Daucus carota* (AAD56581.1), *Arabidopsis thaliana* (NP_194019.1), *Matthiola incana* (AAB82287.1), *Brassica rapa* subsp. *oleifera* (ABY89681.1), *Brassica oleracea* var. *capitata* (AAO73440.1), *Fragaria* x *ananassa* (AFP99287.1), *Malus domestica* (AAZ79374.1), *Pyrus communis* (AGL50919.1), *Prunus persica* (ABX89941.1), *Prunus salicina* var. *cordata* (AEN19292.1), *Prunus avium* (ADZ54785.1), *Citrus sinensis* (AAT02642.1), *Gossypium hirsutum* (ACH56522.1), *Theobroma cacao*

(ADD51356.1), *Medicago truncatula* (XP_003611189.1), *Glycine max* (NP_001240884.1), *Ipomoea batatas* (ADE08370.1), *Ipomoea nil* (BAB71806.1), *Petunia hybrida* (P51092.1), *Nicotiana tabacum* (AFM52334.1), *Solanum tuberosum* (AEJ90548.1).

The out group of F3H isolated from Arabidopsis (AAM65101.1), grape (XP_002267640.1), apple (AAX89397.1), pomegranate (PgF3H-2, this work; Ophir *et al*., 2014) and petunia (AAC49929.1) is presented as a red cluster.
