# Supplementary material for: A "White" Anthocyanin-less Pomegranate (Punica granatum L.) Caused by an Insertion in the Coding Region of the Leucoanthocyanidin Dioxygenase (LDOX; ANS) Gene
Source: PLoS One. 2015 Nov 18;10(11):e0142777. doi: 10.1371/journal.pone.0142777 (PMC4651307; doi:10.1371/journal.pone.0142777)
Supplement: S1 Table — (DOCX) [file pone.0142777.s006.docx]

**S1 Table.** **Primers used for semi-quantitative RT-PCR experiments.**

| **Gene**  **detected** | **GenBank accession number** | **Primer name** | **Sequence (5'→3')** | **Ann. temp*** | **Size pro** (bp)** |
| --- | --- | --- | --- | --- | --- |
| *PgPAL-4* | Ophir *et al*., 2014 | PAL-S-F | CATGGCGGGAACTTCCA | 55 °C | 300 |
|  |  | PAL-S-R | CTGGACGTGGTTCGTCAC |  |  |
| *PgCHS-3* | Ophir *et al*., 2014 | CHS3-F1 | CCCACTAAAGCGACCCATT | 58 °C | 140 |
|  |  | CHS3-R2 | AGACCACAAAATGCCTCCAC |  |  |
| *PgCHI* | Ophir *et al*., 2014 | CHI-S-F2 | GTGAAGTACACAGCAATAGGAGTG | 50 °C | 280 |
|  |  | CHI-S-R1 | CATCCAGGAACCAGTCAATG |  |  |
| *PgF3H-2* | Ophir *et al*., 2014 | F3H-S-F | GTCGCGTACAACCAGTTCA | 55 °C | 320 |
|  |  | F3H-S-R | CAATGAATCCGCCCTTCT |  |  |
| *PgF3'H* | Ophir *et al*., 2014 | F3'H-F2 | GTGGCGGATGTTGAGGAAG | 58 °C | 500 |
|  |  | F3'H-R4 | TTTCGGTATCGGTGAGGTTC |  |  |
| *PgF3'5'H* | Ophir *et al*., 2014 | F3'5'H-S-F2 | GTGGAAGCTGCTGAGGAA | 55 °C | 300 |
|  |  | F3'5'H-S-R2 | ATCCCCAATGTTGAAGAACC |  |  |
| *PgDFR* | JF747150 | DFR-S-F | GACCCTGAGAATGAAGTGATCA | 55 °C | 250 |
|  |  | DFR-S-R2 | CATCCATCCGGTCATCTTG |  |  |
| *PgLDOX* | JF747149 | LDOX-S-F | GAGGAGATGCTGCTGCAG | 55 °C | 280 |
|  |  | LDOX-S-R | CCTCACCTTCTCCTTGTTCAC |  |  |
| *PgAN2*  (MYB) | JF747151 | AN2-S-F1 | CGACCTTCTTCGGAAATGTG | 55 °C | 220 |
|  |  | AN2-S-R2 | GCAATCAACGTCCATCTGT |  |  |
| *PgAN1*  (bHLH) | JF747152 | AN1-S-F | CCGTCGAGAAATGGCTGT | 58 °C | 325 |
|  |  | AN1-S-R | CCATTCTGTCTCGGTCAGGT |  |  |
| *PgWD40* | HQ199314 | TTG1-S-F | GTCTCTGCTGATGGGTCGGT | 60 °C | 350 |
|  |  | TTG1-S-R | CAGCAGAGTACATCGACATTGG |  |  |

*Ann. temp. –annealing temperature.

**Size pro. – size product (bp).
