# Supplementary material for: A "White" Anthocyanin-less Pomegranate (Punica granatum L.) Caused by an Insertion in the Coding Region of the Leucoanthocyanidin Dioxygenase (LDOX; ANS) Gene
Source: PLoS One. 2015 Nov 18;10(11):e0142777. doi: 10.1371/journal.pone.0142777 (PMC4651307; doi:10.1371/journal.pone.0142777)
Supplement: S2 Table — (DOCX) [file pone.0142777.s007.docx]

**S2 Table. List of primers used in this work and their specific role.**

| **Primer name** | **Purpose** | **Sequence (5'→3')** |
| --- | --- | --- |
| PgLDOX-F1 | qRT-PCR analysis | CTGGTCAGGAACTGTCATGG |
| PgLDOX-R1 | qRT-PCR analysis | ACAGCCCAAATATCGACCAA |
| PgRPSΙΙ-F1 | qRT-PCR analysis | TCAATTTGTGAGGGTCGTTCT |
| PgRPSΙΙ-R1 | qRT-PCR analysis | GATTCAAGAGTAGTAACCGATTCCA |
| PgLDOX-F4 | SNP identified | GCTTGTCACTGAGGCAGAG |
| PgLDOX-R3 | SNP identified | CCTAGTGAAGAAAGCTGAGTAGAAG |
| PgLDOX-F2 | primer that flanks the insertion | CTTCACATTCATTCATCACATAG |
| PgLDOX-R2 | primer that flanks the insertion and AFLP analysis | ACTCAATGTCCCTTAGGTCA |
| *MseI* adaptor  (MSEI RP) | AFLP analysis | GACGATGAGTCCTGAGTAA |
| PgLDOX-R5 | AFLP analysis | TCAATGAGGTTGTCAGGGA |
| PgLDOX-R7 | AFLP analysis | ATCACTCGGGATCTTAGGC |
| PgLDOX-F6 | HRM analysis | ATCATACTGAAGCCGCTG |
| PgLDOX-R4 | HRM analysis | TCCTCTGGGTTATTCTTCCT |
| PgLDOX-F18 | primer based on the 18bp of the insertion | GTACTGGTTTCTCATCAACTCAC |
