# Supplementary material for: A "White" Anthocyanin-less Pomegranate (Punica granatum L.) Caused by an Insertion in the Coding Region of the Leucoanthocyanidin Dioxygenase (LDOX; ANS) Gene
Source: PLoS One. 2015 Nov 18;10(11):e0142777. doi: 10.1371/journal.pone.0142777 (PMC4651307; doi:10.1371/journal.pone.0142777)
Supplement: S3 Table — (DOCX) [file pone.0142777.s008.docx]

**S3 Table. Phenotype and genotype segregation analysis of the F2 population.**

| **Generation** | **Name** | **Phenotype** | **SNP** | **Insertion in both alleles of *PgLDOX*** |
| --- | --- | --- | --- | --- |
| "P" | P.G.164-65 | "White" | T/T | + |
|  | 'Nana1' | Red | C/C | - |
| "F1"  (5 out of 79) | 13/1(5) | Red | C/T | - |
|  | 13/16(5) | Red | C/T | - |
|  | **13/35(5)** | Red | C/T | - |
|  | 13/62(5) | Red | C/T | - |
|  | 13/68(5) | Red | C/T | - |
| "F2" –  Self -pollination of 13/35(5).  91 F2 progeny, in total. | 9/1(7) | Red | C/T | - |
|  | 9/2(7) | Red | C/C | - |
|  | 9/4(7) | Red | C/T | - |
|  | 9/5(7) | Red | C/C | - |
|  | 9/6(7) | Red | C/T | - |
|  | 9/9(7) | "White" | T/T | + |
|  | 9/10(7) | Red | C/T | - |
|  | 9/11(7) | Red | C/T | - |
|  | 9/12(7) | Red | C/T | - |
|  | 9/13(7) | "White" | T/T | + |
|  | 9/14(7) | Red | C/T | - |
|  | 9/15(7) | "White" | T/T | + |
|  | 9/16(7) | Red | C/C | - |
|  | 9/17(7) | Red | C/T | - |
|  | 9/18(7) | Red | C/T | - |
|  | 9/19(7) | Red | C/C | - |
|  | 9/20(7) | "White" | T/T | + |
|  | 9/21(7) | Red | C/T | - |
|  | 9/22(7) | Red | C/C | - |
|  | 9/23(7) | Red | C/T | - |
|  | 9/24(7) | Red | C/T | - |
|  | 9/25(7) | Red | C/T | - |
|  | 9/26(7) | Red | C/C | - |
|  | 9/27(7) | "White" | T/T | + |
|  | 9/28(7) | Red | C/C | - |
|  | 9/29(7) | Red | C/C | - |
|  | 9/30(7) | Red | C/C | - |
|  | **Name** | **Phenotype** | **SNP** | **Insertion in both alleles of *PgLDOX*** |
|  | 9/31(7) | Red | C/T | - |
|  | 9/32(7) | "White" | T/T | + |
|  | 9/33(7) | Red | C/T | - |
|  | 9/34(7) | Red | C/T | - |
|  | 9/35(7) | Red | C/T | - |
|  | 9/36(7) | "White" | T/T | + |
|  | 9/37(7) | Red | C/T | - |
|  | 9/38(7) | "White" | T/T | + |
|  | 9/39(7) | Red | C/T | - |
|  | 9/40(7) | "White" | T/T | + |
|  | 9/42(7) | "White" | T/T | + |
|  | 9/43(7) | Red | C/T | - |
|  | 9/44(7) | Red | C/T | - |
|  | 9/45(7) | "White" | T/T | + |
|  | 9/46(7) | "White" | T/T | + |
|  | 9/47(7) | Red | C/C | - |
|  | 9/48(7) | Red | C/T | - |
|  | 9/49(7) | "White" | T/T | + |
|  | 9/50(7) | Red | C/T | - |
|  | 9/51(7) | Red | C/C | - |
|  | 9/52(7) | Red | C/C | - |
|  | 9/53(7) | Red | C/T | - |
|  | 9/56(7) | Red | C/C | - |
|  | 9/57(7) | Red | C/T | - |
|  | 9/59(7) | "White" | T/T | + |
|  | 9/60(7) | Red | C/C | - |
|  | 9/61(7) | Red | C/T | - |
|  | 9/62(7) | "White" | T/T | + |
|  | 9/63(7) | Red | C/T | - |
|  | 9/64(7) | "White" | T/T | + |
|  | 9/65(7) | Red | C/C | - |
|  | 9/66(7) | "White" | T/T | + |
|  | 9/67(7) | Red | C/C | - |
|  | 9/68(7) | Red | C/T | - |
|  | 9/69(7) | Red | C/C | - |
|  | 9/70(7) | Red | C/T | - |
|  | 9/71(7) | Red | C/T | - |
|  | 9/72(7) | Red | C/T | - |
|  | 9/73(7) | Red | C/T | - |
|  | 9/74(7) | "White" | T/T | + |
|  | **Name** | **Phenotype** | **SNP** | **Insertion in both alleles of *PgLDOX*** |
|  | 9/75(7) | Red | C/C | - |
|  | 9/76(7) | Red | C/T | - |
|  | 9/77(7) | Red | C/C | - |
|  | 9/78(7) | Red | C/T | - |
|  | 9/79(7) | Red | C/T | - |
|  | 9/80(7) | Red | C/C | - |
|  | 9/81(7) | Red | C/T | - |
|  | 9/82(7) | Red | C/T | - |
|  | 9/83(7) | Red | C/C | - |
|  | 9/84(7) | Red | C/T | - |
|  | 9/85(7) | Red | C/T | - |
|  | 9/86(7) | "White" | T/T | + |
|  | 9/87(7) | Red | C/T | - |
|  | 9/88(7) | Red | C/T | - |
|  | 9/89(7) | Red | C/T | - |
|  | 9/90(7) | "White" | T/T | + |
|  | 9/91(7) | "White" | T/T | + |
|  | 9/92(7) | Red | C/T | - |
|  | 9/93(7) | "White" | T/T | + |
|  | 9/94(7) | Red | C/T | - |
|  | 9/95(7) | "White" | T/T | + |
|  | 9/96(7) | Red | C/C | - |
|  | 9/97(7) | "White" | T/T | + |
|  | 9/98(7) | Red | C/T | - |

In summary,

| **F2 progeny** | **Expected** | **Observed** |
| --- | --- | --- |
| "White" homozygote- (T/T) | 25%=22.75 | 24 |
| Red homozygote- (C/C) | 25%=22.75 | 22 |
| Red Heterozygote- (C/T) | 50%=45.5 | 45 |
| **Total** | **91** | **91** |
